# Phenolic Constituents of Chinese Quince Twigs (*Chaenomeles sinensis* Koehne) and Their Anti-Neuroinflammatory, Neurotrophic, and Cytotoxic Activities

**DOI:** 10.3390/antiox10040551

**Published:** 2021-04-02

**Authors:** Dong Hyun Kim, Lalita Subedi, Hye Ryeong Kim, Sang Un Choi, Sun Yeou Kim, Chung Sub Kim

**Affiliations:** 1 School of Pharmacy, Sungkyunkwan University, Suwon 16419, Korea; eodrnduf1@skku.edu (D.H.K.); khryoung@skku.edu (H.R.K.); 2 Gachon Institute of Pharmaceutical Science, Gachon University, Incheon 21936, Korea; subedilali@gmail.com (L.S.); sunnykim@gachon.ac.kr (S.Y.K.); 3 Korea Research Institute of Chemical Technology, Daejeon 34114, Korea; suchoi@krict.re.kr; 4 College of Pharmacy, Gachon University, #191, Hambakmoero, Yeonsu-gu, Incheon 21936, Korea; 5 Department of Biopharmaceutical Convergence, Sungkyunkwan University, Suwon 16419, Korea

**Keywords:** *Chaenomeles sinensis*, Rosaceae, phenolic constituents, anti-inflammation, neurotrophic effect, cytotoxicity

## Abstract

*Chaenomeles sinensis* has been used as a food and traditional medicines. However, most of research on discovering bioactive constituents from this plant have been focused on its yellow fruit, Chinese quince, due to its wide usage. Here, we isolated and characterized three new phenolic compounds (**1**, **9**, and **11**) and 21 known compounds (**2**−**8**, **10**, and **12**−**24**) from the twigs of *C. sinensis*. Their chemical structures were established by spectroscopic and spectrometric data analysis including 1D and 2D NMR, high-resolution mass spectrometry (HRMS), electronic circular dichroism (ECD), and LC-MS analysis. Some of the isolated compounds (**1**−**24**) showed anti-neuroinflammatory effects on nitric oxide (NO) production in lipopolysaccharide (LPS)-activated BV-2 cells, neurotrophic activity in C6 cells through the secretion of nerve growth factor (NGF) and/or cytotoxicity against four human cancer cell lines (A549, SK-OV-3, SK-MEL-2, MKN-1).

## 1. Introduction

Phenolic compounds are one of the largest natural products groups, especially in the plant kingdom, and are characterized by the presence of at least a benzene ring with hydroxy group(s) attached to it [1]. They can be classified into several subgroups according to the number of carbon atoms and their arrangement as follows: phenolic acids (C_6_–C_1_), acetophenones and phenylacetic acids (C_6_–C_2_), phenylpropanoids (C_6_–C_3_), naphthoquinones (C_6_–C_4_), xanthones (C_6_–C_1_–C_6_), stilbenoids (C_6_–C_2_–C_6_), flavonoids (C_6_–C_3_–C_6_), and lignans (C_6_–C_3_–C_3_–C_6_) [1,2]. Many of these well-known natural products in the phenolic compounds class have been reported to possess diverse pharmacological activities including anti-inflammatory (e.g., salicylic acid), neurotrophic (e.g., 6-shogaol) [2,3], antioxidant (e.g., resveratrol, quercetin) [2], and anticancer (e.g., podophyllotoxin) [2] effects.

Neurodegenerative diseases such as dementia and Parkinson’s disease are exploding due to rapid aging populations [4]. Despite various efforts to overcome these neurodegenerative diseases, there are not sufficient reports on drugs that modify the disease [5]. Therefore, it is very important to draw meaningful leading compounds from natural resources in order to regulate neurodegenerative diseases. As neurodegenerative diseases are highly complex and incurable things, there are some aspects that make it difficult to overcome diseases with a single target such as amyloid beta [5]. Thus, we should think about the study of exploring phytochemicals that overcome neurodegenerative diseases by regulating multiple targets. In this study, the effects of phytochemicals with anti-neuroinflammation and neurotrophic factor potentiation will be shown. They are important multi-targets that cause the induction and progression of neurodegenerative diseases.

**Figure 1:**
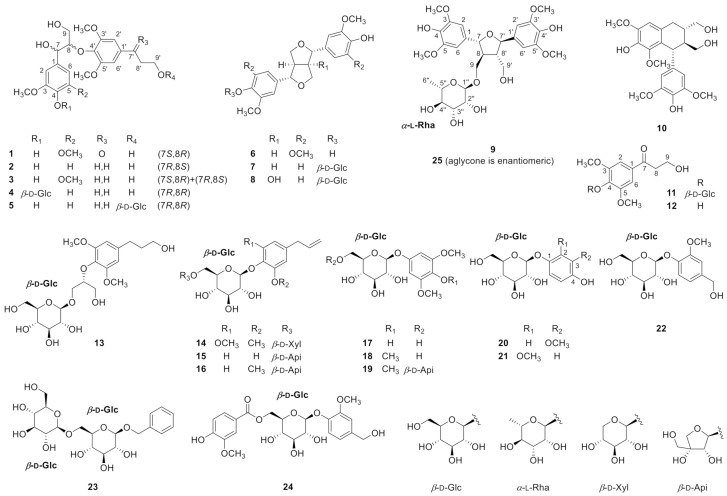
Chemical structure of compounds **1**–**25**.

*Chaenomeles sinensis* Koehne (or *Pseudocydonia sinensis*, Chinese Quince, Rosaceae) is a semi-evergreen tree in the family Rosaceae and is widely distributed in East Asia, including Korea, mainland China, and Japan. The yellow fruit of this plant has been consumed as a tea, in a candied form, or in liquor [6], and also used as a Korean traditional medicine over 400 years to treat myalgia, beriberi, vomiting, and diarrhea [7]. Due to the wide range of usages of *C. sinensis* fruit, the phytochemical and biological research on this plant has been mainly focused on the fruit [8,9,10,11,12,13] rather than the other parts such as twigs [14,15] or leaves. Based on these previous studies, it was found that the major compound classes of *C. sinensis* were triterpenoids [9,14,15,16] and flavonoids [8,13,15]. However, minor components such as non-flavonoid phenolic compounds in the *C. sinensis* twigs with anti-neurodegerative effects remain largely unknown. In this study, 24 phenolic compounds (**1–24**) including three new compounds (**1**, **9**, and **11**) were isolated and characterized from the twigs of *C. sinensis* (Figure 1). Their structures were elucidated by conventional NMR techniques coupled with simulation of ^1^H NMR peak, analysis of HRMS and ECD data, and hydrolysis followed by LC-MS analysis. The isolates (**1**−**24**) were tested for their anti-neuroinflammatory, neurotrophic, and cytotoxic activities, and herein, we report the isolation and structure elucidation of phenolic phytochemicals from the twigs of *C. sinensis* as well as their biological activities.

## 2. Materials and Methods

### 2.1. General Experimental Procedures

JASCO P-1020 polarimeter equipped with the sodium D line (590 nm) (JASCO, Easton, MD, USA) was performed to measure specific rotations. Bruker AVANCE III 700 NMR spectrometer at 700 MHz (^1^H) and 175 MHz (^13^C) was carried out to measure the NMR spectra (^1^H, ^13^C, COSY, HSQC, and HMBC) at 700 MHz (^1^H) and 175 MHz (^13^C) with chemical shifts given in ppm (δ) (Bruker, Karlsruhe, Germany). High-resolution fast atom bombardment mass spectroscopy (HRFABMS) were measured on either a Waters SYNAPT G2 (Milford, MA, USA) or a JEOL JMS700 mass spectrometer (Tokyo, Japan). LC-MS data were measured using an Agilent 1290 Infinity II HPLC instrument (Foster City, CA, USA) coupled to a G6545B quadrupole time-of-flight (Q-TOF) mass spectrometer (Agilent Technologies) with a Kinetex C_18_ 5 µm column (250 mm length × 4.6 mm i.d.; Phenomenex, Torrance, CA, USA). The semipreparative high-performance liquid chromatography (HPLC) furnished with a Gilson 306 pump (Middleton, WI, USA) and a Shodex refractive index detector (New York, NY, USA) was used. Column chromatography was performed employing either silica gel 60 (70−230 and 230−400 mesh; Merck, Darmstadt, Germany) or RP-C_18_ silica gel (Merck, 230−400 mesh). Sephadex LH-20 (Pharmacia, Uppsala, Sweden) was utilized for molecular sieve column chromatography. Merck precoated silica gel F_254_ plates and RP-C_18_ F_254s_ plates (Merck, Darmstadt, Germany) were used for thin-layer chromatography (TLC) analysis. Spots were detected on TLC under UV light or by heating after spraying with anisaldehyde−sulfuric acid.

### 2.2. Plant Material

Twigs of two-year-old *C. sinensis* (7.0 kg) were purchased at Yangjae Flower Market in Seoul, Korea, in January 2012. A voucher specimen of the plants (SKKU-NPL 1206) was authenticated by Prof. Kang Ro Lee and deposited in the herbarium of the School of Pharmacy, Sungkyunkwan University, Suwon, Korea.

### 2.3. Extraction and Isolation

Twigs of *C. sinensis* (7.0 kg) were extracted with 80% aqueous MeOH (each 10 L × 1 day, 3 times) under reflux and filtered. The filtrate was evaporated in vacuo to yield a MeOH extract (320 g). The 80% MeOH extract was suspended in distilled water and successively partitioned with *n*-hexane (3 g), CHCl_3_ (15 g), EtOAc (6 g), and *n*-BuOH (30 g). Repeated column chromatographical fractionation and isolation of CHCl_3_, EtOAc, and *n*-BuOH soluble layers afforded the compounds **1**–**24**. (See Supplementary Materials for the detailed isolation process).

*(7S,8R)-4,7,9,9′-tetrahydroxy-3,5,3′,5′-tetramethoxy-7′-oxo-8-O-4′-neolignan (**1**)*. Colorless gum; [α]${}_{D}^{25}$−12 (*c* 0.1, MeOH); ECD (MeOH) *λ*_max_ (Δ*ε*) 244 (−2.3) nm; ^1^H and ^13^C NMR data, see Table 1; HRFABMS *m/z* 451.1600 [M − H]^−^ (calcd for C_22_H_27_O_10_, 451.1599) (Figures S1–S7).

*(7R,8S)-4,7,9,9′-tetrahydroxy-3,3′,5′-trimethoxy-8-O-4′-neolignan (**2**)*. Colorless gum; ECD (MeOH) *λ*_max_ (Δ*ε*) 241 (+2.1) nm; ^1^H and ^13^C NMR data, see Table 1.

1:1 mixture of (7S,8R)- and (7R,8S)-4,7,9,9*′*-tetrahydroxy-3,5,3′,5′-tetramethoxy-8-O-4*′*-neolignan (**3**). Colorless gum; ^1^H and ^13^C NMR data, see Table 1.

*7R,7′R,8S,8′S-icariol A2 9′-O-α-l-rhamnopyranoside (**9**)*. Colorless gum; [α]${}_{D}^{25}$+30 (*c* 0.1, MeOH); ECD (MeOH) *λ*_max_ (Δ*ε*) 246 (+5.3), 208 (−10.1) nm; ^1^H and ^13^C NMR data, see Table 1; HRFABMS *m/z* 581.2231 [M − H]^−^ (calcd for C_28_H_37_O_13_, 581.2229) (Figures S8–S15).

*β-hydroxypropiosyringone 4′-O-β-d-glucopyranoside (**11**)*. Colorless gum; [α]${}_{D}^{25}$+24 (*c* 0.25, MeOH); ^1^H and ^13^C NMR data, see Table 2; HRFABMS *m/z* 411.1263 [M + Na]^+^ (calcd for C_17_H_24_O_10_Na, 411.1262) (Figures S16–S22).

### 2.4. ^1^H NMR Simulation for H-7 of Compound **1**

MestReNova Version 14.1.2-25024 was used to simulate the H-7 peak. The “Spin Simulation” under the “Prediction” tab was clicked to open the dialog box. The “Spin Groups” was set to be “2” (default value), the “Shifts (ppm)” values of “A” and “B” were given “4.987” and “4.232”, respectively, with the “Line Width (Hz)” of both “3.0”. After the coupling constant between “A” and “B” was set as “3.5” or “6.7”, the icon with “New Simulation” at the top left was clicked to generate the simulated ^1^H NMR peaks of H-7 and H-8 of **1**.

### 2.5. Acid Hydrolysis of Compounds **6** and **11** and Sugar Analysis

The sugar analysis experiment was performed as described in previous communication by Tanaka et al. with some modifications [17]. Compounds **9** and **11** (each of 0.5 mg) were individually hydrolyzed with 1 N HCl (1.0 mL) for 2 h at 100 °C. Each hydrolysate was extracted using CHCl_3_, and then the aqueous layers were evaporated to remove the HCl and to afford sugar from each hydrolysate. Each H_2_O-soluble fraction was added to anhydrous pyridine (400 µL) containing l-cysteine methyl ester hydrochloride (1.1 mg), which were heated at 60 °C for 1 h. Then, *O*-tolyl isothiocyanate (30 µL) was added to the reaction mixture and heated at 60 °C for another 1 h. After the reaction, each reaction mixture was analyzed by LC-MS (0.7 mL/min; 25% aqueous CH_3_CN with 0.1% formic acid for 40 min). The authentic samples of l-rhamnopyranose and d-glucopyranose were derivatized and analyzed by the same method mentioned above, and these monosaccharides were also derivatized with d-cysteine methyl ester hydrochloride instead of its l-form to prepare their enantiomeric derivatives. The hydrolysate derivatives of **9** and **11** were 25.4 min (l-rhamnopyranose) and 14.9 min (d-glucopyranose) which matched well with those of authentic l-rhamnopyranose (25.3 min) and d-glucopyranose (14.9 min), respectively, rather than their enantiomeric derivatives (13.2 min (d-rhamnopyranose) and 13.7 min (l-glucopyranose)) (Figures S15 and S22).

### 2.6. NO Production Assays

Analogous as described in [18]. The BV-2 cells, developed by Dr. V. Bocchini at the University of Perugia (Perugia, Italy), were used for this study [19,20]. The cells were seeded in a 96-well plate (4 × 10^4^ cells/well) and incubated in the presence or absence of various doses of tested compounds (**1**–**24**). Lipopolysaccharide (LPS) (100 ng/mL) was added to all the pre-treated wells except the control one and grown for 1 d. The produced levels of nitrite (NO_2_), a soluble oxidized product of NO, was evaluated with 0.1% *N*-1-napthylethylenediamine dihydrochloride and 1% sulfanilamide in 5% phosphoric acid, aka the Griess reagent. The supernatant (50 μL) was mixed with the Griess reagent (50 μL). After 10 min the absorbance was gauged at 570 nm. For a positive control, the reported nitric oxide synthase (NOS) inhibitor *N*^G^-monomethyl-l-arginine (l-NMMA) was employed. Graded sodium nitrite solution was utilized to determine nitrite concentrations. A 3-[4,5-dimethylthiazol-2-yl]-2,5-diphenyltetrazolium bromide (MTT) assay was used for the cell viability assay.

### 2.7. Assays for Nerve Growth Factor NGF Release from C6 Cells

Analogous as described in [21]. C6 glioma cell lines were used to measure the nerve growth factor (NGF) of the culture medium, which were fixed with 10% fetal bovine serum (FBS) and 1% penicillin-streptomycin (PS) in an incubator filled with 5% CO_2_. The cells were seeded in a 24-well culture plate (1 × 10^5^ cells/well) and incubated for 24 h. The cells were treated with or without 20 µM of the compounds (**1**–**24**), together with serum-free Dulbecco’s modified Eagle’s medium (DMEM) for another 24 h. Released NGF levels from the supernatants from each cell were measured using an ELISA development kit (R&D System, Minneapolis, MN, USA). Besides, the cell viability was evaluated by 3-[4,5-dimethylthiazol-2-yl]-2,5-diphenyltetrazolium bromide (MTT) assay by comparison with 6-shogaol as a positive control and the results are expressed as percentage of the control group.

### 2.8. Cytotoxicity Assessment

The cytotoxicity of purified compounds (**1**–**24**) were tested against the A-549 (non-small cell lung adenocarcinoma), SK-OV-3 (ovary malignant ascites), SK-MEL-2 (skin melanoma), and MKN-1 (adenosquamous carcinoma), utilizing the sulforhodamine B colorimetric (SRB) method [22]. Cisplatin (≥98%; Sigma–Aldrich) served as a positive control.

## 3. Results and Discussion

### 3.1. Structure Elucidation of Compounds **1**–**24**

**Figure 2:**
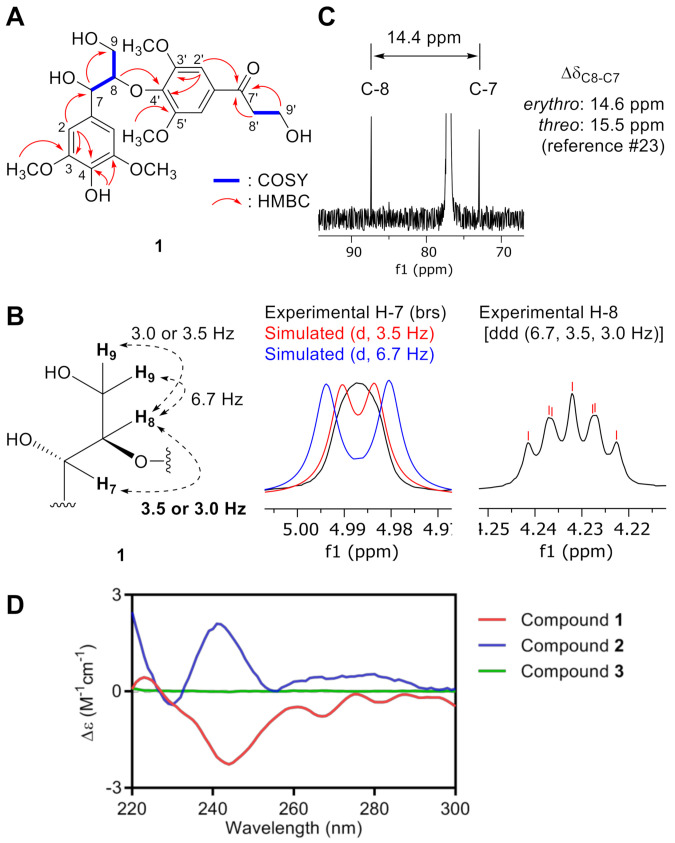
Structure elucidation of **1–3**. (**A**) Key COSY and HMBC correlations of **1**. (**B**) Coupling constants values among H-7, H-8, and H-9 (left) deduced by analysis of ^1^H NMR peaks of H-7 (middle) and H-8 (right). (**C**) Zoomed-in ^13^C NMR spectra of **1** for C-7 and C-8. (**D**) ECD spectra of **1–3**. The negative or positive Cotton effect around 240–245 nm indicated the 8*R*- or 8*S*-configuration, respectively.

Compound **1** was purified as a colorless gum and its molecular formula was determined as C_22_H_28_O_10_ according to the deprotonated molecular ion at *m*/*z* at 451.1600 [M – H]^–^ in the HRFABMS data (calcd. for C_22_H_27_O_10_, 451.1599). Analysis of the ^1^H NMR spectrum of **1** indicated the presence of two 1,3,4,5-tetrasubstituted benzene rings (*δ*_H_ 7.29 (2H, s, H-2′,6′) and 6.58 (2H, s, H-2,6)), two oxygenated methines (*δ*_H_ 4.99 (1H, brs, H-7) and 4.23 (1H, ddd, *J* = 6.6, 3.5, 3.0 Hz, H-8)), two oxygenated methylenes (*δ*_H_ 4.06 (2H, t, *J* = 5.3 Hz, H-9′), 3.93 (1H, overlap, H-9a), and 3.54 (1H, m, H-9b)), a methylene (*δ*_H_ 3.23 (2H, t, *J* = 5.4 Hz, H-8′)), and four methoxy groups (*δ*_H_ 3.96 (6H, s, 3′,5′-OCH_3_) and 3.88 (6H, s, 3,5-OCH_3_)). The ^13^C spectrum of **1** showed 22 carbon signals for two 1,3,4,5-tetrasubstituted benzene rings (*δ*_C_ 153.6 (×2), 147.2 (×2), 139.9, 134.2, 133.1, 130.3, 105.6 (×2), and 102.5 (×2)), a carbonyl carbon (*δ*_C_ 199.1), four methoxy carbons (*δ*_C_ 56.6 (×2) and 56.5 (×2)), four oxygenated carbons (*δ*_C_ 87.5, 73.1, 60.7, and 58.3), and a methylene carbon (*δ*_C_ 40.4). These NMR spectra of **1** were similar to those of **3** (Table 1) [23], except for the presence of the carbonyl carbon signal at *δ*_C_ 199.1 (C-7′) in **1** rather than the methylene carbon signal at *δ*_C_ 34.4 (C-7′) in **3**. The HMBC correlations from H-2′,6′/H-8′/H-9′ to C-7′ were observed, suggesting that carbonyl carbon is located at C-7′. The planar structure of **1** was established through 2D NMR spectra analysis including COSY, HSQC, and HMBC spectra (Figure 2A). In 8-*O*-4′-oxyneolignan derivatives, the relatively small (~4 Hz) and large (~8 Hz) coupling constants between H-7 and H-8 indicate *erythro*- or *threo*-configuration, respectively, in chloroform-*d* [24,25,26]. When the H-7 peak was analyzed to measure the ^3^*J*_H-7,H-8_ value, however, its shape was found to be a broad singlet. Alternatively, the H-8 peak showed the splitting pattern of doublet of doublet of doublet (ddd) with coupling constants of 6.7, 3.5, and 3.0 Hz. In order to deduce the ^3^*J*_H-7,H-8_ value among them, the H-7 peak was simulated with coupling constants of 3.5 and 6.7 Hz. As shown in Figure 2B (middle) the simulated peak with the 3.5 Hz coupling constant was more similar to that of experimental peak rather than that with 6.7 Hz coupling constant. Therefore, the ^3^*J*_H-7,H-8_ value was assigned as 3.0 or 3.5 Hz which suggested *erythro*-form. The difference of ^13^C NMR chemical shift between C-7 and C-8 can be also used for its relative configuration assignment. The Δδ_C-8,C-7_ value was ~14.6 ppm in the *erythro*-form whereas ~15.5 ppm was observed in the *threo*-form [24]. By observing 14.4 ppm of Δδ_C-8,C-7_ value in the ^13^C NMR data of **1** (Figure 2C), the relative configuration between C-7 and C-8 was deduced as *erythro*-form. The 8*R* configuration of **1** was confirmed by means of analysis of its ECD spectrum displaying a characteristic negative Cotton effect at 244 nm (Figure 2D) [26,27]. Thus, the structure of **1** was established as (7*S*,8*R*)-7,9,9′-trihydroxy-3,5,3′,5′-tetramethoxy-7′-oxo-8-*O*-4′-neolignan.

Compounds **2** and **3** were previously reported from *Bursera tonkinensis* [23], and *Epimedium sagittatum* [28] and *Fraxinus mandshurica* [29], respectively, without determination of the absolute configuration of C-7 and C-8 (Figure S23–S26). The relative configuration between C-7 and C-8 were assigned as *erythro* by the same empirical rule applied for **1**; the relatively small ^3^*J*_H-7,H-8_ value was observed from both **2** (3.5 Hz) and **3** (3.2 Hz) (Table 1). A positive Cotton effect at 241 nm from ECD spectrum of **2** indicated an 8*S* configuration of **2** and the absence of any characteristic Cotton effect from the ECD spectrum of **3**, suggesting **3** as a racemic mixture (Figure 2D).

**Figure 3:**
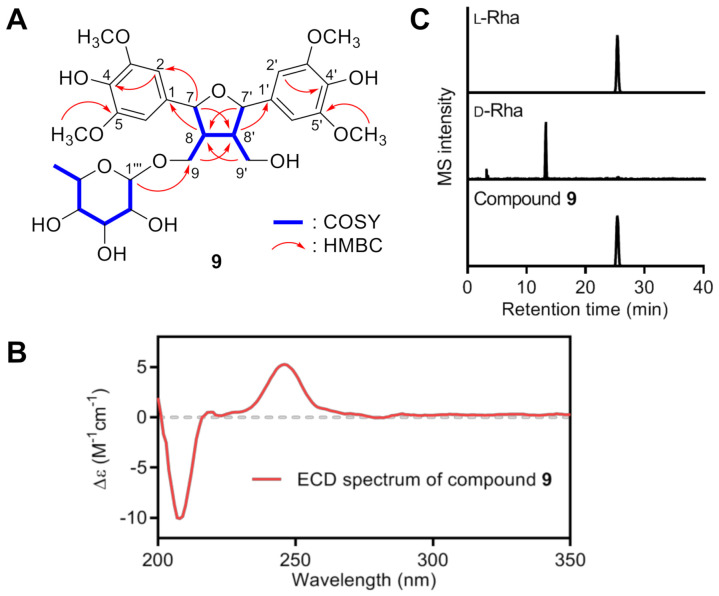
Structure elucidation of **9**. (**A**) Key COSY and HMBC correlations of **9**. (**B**) Extracted ion chromatograms (EICs, *m*/*z* 431.1311) of chiral derivatized l- and d-rhamnopyranose purchased or obtained by hydrolysis of **9**. (**C**) ECD spectrum of **9**. The positive Cotton effect at 246 nm indicated the 7*R*,7′*R*,8*S*,8′*S*-configuration.

Compound **9** was isolated as a colorless gum with a molecular formula of C_28_H_38_O_13_. The ^1^H NMR spectrum of **9** showed two 1,3,4,5-tetrasubstituted benzene rings (*δ*_H_ 6.76 (2H, s, H-2′,6′) and 6.75 (2H, s, H-2,6)), two oxygenated methines (*δ*_H_ 5.06 (1H, d, *J* = 8.4 Hz, H-7′) and 5.03 (1H, d, *J* = 8.4 Hz, H-7)), two oxygenated methylenes (*δ*_H_ 3.82 (1H, m, H-9a), 3.78 (1H, dd, *J* = 11.2, 5.2 Hz, H-9′a), 3.69 (1H, dd, *J* = 11.2, 4.4 Hz, H-9′b), and 3.57 (1H, dd, *J* = 9.9, 4.9 Hz, H-9′b)), two methines (2.51 (1H, m, H-8) and 2.38 (1H, m, H-8′)), four methoxy groups (*δ*_H_ 3.90 (12H, s, 3,5,3′,5′-OCH_3_)), and an *α*-rhamnopyranosyl unit (*δ*_H_ 4.70 (1H, d, *J* = 1.3 Hz, H-1′′), 3.81 (1H, m, H-2′′), 3.65 (1H, dd, *J* = 4.5, 3.2 Hz, H-3′′), 3.61 (1H, dq, *J* = 9.5, 6.5 Hz, H-5′′), 3.40 (1H, m, H-4′′), and 1.27 (3H, d, *J* = 6.5 Hz, H-6′′)). The ^13^C NMR spectrum of **2** revealed 28 resonances, comprising 12 benzene ring carbons [*δ*_C_ 149.5 (×4), 136.4, 136.3, 134.7, 134.3, 105.1 (×2), and 104.8 (×2)], four methoxy carbons (*δ*_C_ 57.0 (×4)), four oxygenated carbons (*δ*_C_ 85.2, 84.5, 67.8, and 61.6), two methine carbons (*δ*_C_ 54.6 and 51.8), and a rhamnopyranosyl unit (*δ*_C_ 102.3, 74.1, 72.7, 72.3, 70.3, and 18.2). These spectroscopic data were almost identical with those of 7*S*,7′*S*,8*R*,8′*R*-icariol A2 9′-*O*-α-l-rhamnopyranoside (**25**) [30]. Intensive 2D NMR data analysis (Figure 3A) confirmed that **9** and **25** have the same the planar structures. The absolute configuration of the known compound **25** was determined as 7*S*,7′*S*,8*R*,8′*R* by observing the negative Cotton effect around 240–250 nm in the ECD spectrum (Figure S9) [30], however, the new compound **9** showed the positive Cotton effect around that area (246 nm, Figure 3B), indicating 7*R*,7′*R*,8*S*,8′*S*-configuration. In addition, the opposite sign of the specific rotation values of **9,** [α]${}_{D}^{25}$+ 30 (*c* 0.1, MeOH), and **25**, [α]${}_{D}^{25}$− 51.4 (*c* 0.1, MeOH) [30], supported the initial assignment. Finally, the l-form of rhamnopyranosyl moiety was assigned by LC-MS analysis on the chiral derivatized monosaccharide obtained by hydrolysis of **9** and following chemical reaction [17]. The retention time of derivatized rhamnopyranose (25.4 min) was matched with that of standard l-rhamnopyranose (25.3 min) rather than d-rhamnopyranose (13.2 min) (Figure 2C). Thus, the structure of **9** was determined as 7*R*,7′*R*,8*S*,8′*S*-icariol A2 9′-*O*-α-l-rhamnopyranoside.

**Figure 4:**
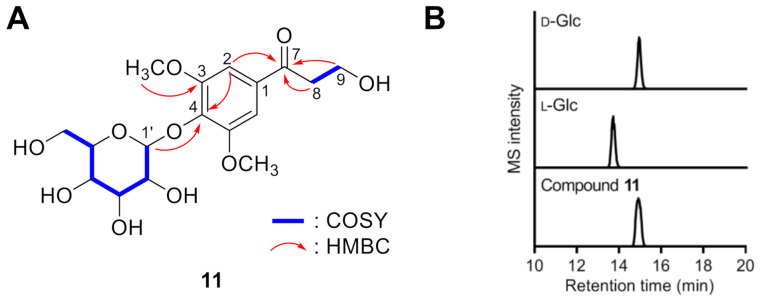
Structure elucidation of **11**. (**A**) Key COSY and HMBC correlations of **11**. (**B**) Extracted ion chromatograms (EICs, *m*/*z* 447.1260) of chiral derivatized d- and l-glucopyranose purchased or obtained by hydrolysis of **11**.

Compound **11** was obtained as a colorless gum, and its molecular formula was deduced as C_17_H_24_O_10_ based upon the HRFABMS data. The ^1^H and ^13^C NMR spectra of **11** were comparable to those of 3-hydroxyl-1-(4-hydroxy-3,5-dimethoxyphenyl)-1-propanone (or *β*-hydroxypropiosyringone, **12**) [31], except for the presence of resonances for a *β*-glucopyranosyl unit (*δ*_H_ 5.00 (1H, d, *J* = 7.5 Hz, H-1′), 3.67 (1H, dd, *J* = 11.9, 2.3 Hz, H-6′a), 3.55 (1H, dd, *J* = 11.9, 5.3 Hz, H-6′b), 3.40 (1H, dd, *J* = 8.8, 7.5 Hz, H-2′), 3.33 (1H, t, *J* = 8.8 Hz, H-3′), 3.30 (1H, t, *J* = 8.8 Hz, H-4′), 3.12 (1H, ddd, *J* = 8.8, 5.3, 2.3 Hz, H-5′); *δ*_C_ 104.6, 78.6, 78.0, 75.9, 71.5, and 62.7). On the basis of the HMBC cross peak of H-1′ with C-4, the location of the *β*-glucopyranosyl unit was confirmed to be connected at C-4 by an ether bond (Figure 4A). Acid hydrolysis of **11** afforded glucopyranose, which was identified as d-form by the same method as described above (Figure 4B). Thus, the structure of **11** was elucidated as *β*-hydroxypropiosyringone 4′-*O*-*β*-d-glucopyranoside.

The other known compounds were identified as (7*S*,8*R*)-1-[4-(O-*β*-d-glucopyranosyl)-3-methoxyphenyl]-2-[4-(3-hydroxypropyl)-2,6-dimethoxyphenoxyl]-1,3-propanediol (**4**) [32], 3-[4-[(1*R*,2*S*)-2-hydroxy-2-(4-hydroxy-3-methoxyphenyl)-1-(hydroxymethyl)ethoxy]-3-methoxyphenyl]propyl *β*-d-glucopyranoside (**5**) [27], syringatesinol (**6**) [33], pinoresinol-4-*O*-glucoside (**7**) [34], 8-hydroxypinoresinol-4′-*O*-*β*-d-glucose (**8**) [35], (–)-lyoniresinol 3*α*-*O*-*β*-d-glucopyranoside (**10**) [36], 3-hydroxyl-1-(4-hydroxy-3,5-dimethoxyphenyl)-1-propanone (**12**) [31], (2*R*)-*O*-[4′-(3″-hydroxypropyl)-2′,6′-dimethoxyphenyl]-3-*O*-*β*-d-glucopyranosyl-*sn*-glycerol (**13**) [37], 4-allyl-2,6-dimethoxyphenyl 1-*O*-*β*-d-xylopyranosyl-(1→6)-*β*-d-glucopyranoside (**14**) [38], 4-allyl-2-hydroxyphenyl 1-*O*-*β*-d-apiosyl-(1→6)-*β*-d-glucoside (**15**) [39], 4-allyl-2-methoxyphenyl 6-*O*-*β*-d-apiosyl-(1→6)-*β*-d-glucoside (**16**) [40], 3,5-dimethoxy-1-hydroxy-4-*O*-*β*-d-glucoside (**17**) [41], 3,4,5-trimethoxyphenol 1-*O*-*β*-d-glucopyranoside (**18**) [42], 3,4,5-trimethoxyphenyl 1-O-*β*-d-apiofuranosyl-(1′′→6′)-*β*-d-glucoside (**19**) [43], tachioside (**20**), isotachioside (**21**) [44], vanillyl alcohol-4-*O*-*β*-d-glucopyranoside (**22**) [45], benzyl *β*-d-xylopyranosyl-(1→6)-*β*-d-glucopyranoside (**23**) [46], and 4-hydroxy-2,6-dimethoxyphenyl 6′-*O*-vanilloyl-*β*-d-glucopyranoside (**24**) [47] by comparing their spectroscopic data with reported data.

### 3.2. Anti-Neuroinflammatory Activity of the Isolated Compounds (**1**–**24**)

Alzheimer’s disease, a neurodegenerative disease, is an unsolved social and medical problem and is strongly associated with neurotrophins (e.g., NGF, brain-derived neurotrophic factor (BDNF), neurotrohpin-3 (NT-3), and NT-4/5) and neuroinflammatory mediators (e.g., nitric oxide (NO), tumor necrosis factor alpha (TNF-*α*), interleukin (IL)-1*β*, nuclear factor kappa B (NF-κB), and prostaglandin (PG)E_2_) [48]. Therefore, many phytochemicals have been tested to discover novel anti-neurodegenerative agents, which revealed that phenolic constituents are one of the major neurotrophic and anti-neuroinflammatory compounds [48,49]. In line with this finding, the isolated phenolic compounds in this study (**1**−**24**) were assessed for their anti-neuroinflammatory activities by measuring NO levels in LPS-activated BV-2 cells (Table 3) and for their neurotrophic effects evaluated by assessing the secretion of NGF from C6 cells (Table 4). Compounds **1**−**3**, 8–*O*–4′ type neolignans, significantly reduced NO levels with IC_50_ values of 18.63–19.75 μM, which showed better activity than a well-known inhibitor of NO synthase (NOS), *N*^G^-monomethyl-l-arginine (l-NMMA, IC_50_ 21.35 μM) [50]. These data were consistent with the previous studies in which diverse 8–*O*–4′ type neolignans exhibited potent NO inhibitory activity [49]. However, compounds **4** and **5**, the other 8–*O*–4′ type neolignans, were not active (>100 μM) in this study, which might be attributed to the different configuration at C-7 and/or the glucopyranosyl unit attached to the molecules. The most potent compound in this study was **6**, a furanofuran-type lignan, with IC_50_ value of 12.69 μM, whereas the other glucosylated furanofuran-type lignans **7** and **8** did not display NO inhibitory activity. The simple phenolic glucoside **21** was a potent NO inhibitor (IC_50_ 15.00 μM) but transfer of the methoxy group at C-3 to C-2 decreased the potency (IC_50_ 41.67 μM, **20**). The other compounds showed weak or no activity.

### 3.3. Neurotrophic Activity of the Isolated Compounds (**1**–**24**)

Similar to the NO inhibitory activity profile of the 8–*O*–4′ type neolignans, only **1**–**3** displayed effect on NGF release with stimulation levels of 110.27 ± 2.18%, 127.98 ± 3.95%, and 149.89 ± 1.97%, respectively, while the latter was comparable to that of positive control, 6-shogaol (149.53 ± 5.36%) [51]. The addition of a ketone group at C-7′ (**1**) and removal of the methoxy group at C-5 (**2**) in **3** decreased NGF secretion activity. Compound **20** showed powerful NGF release effect with stimulation levels of 157.73 ± 1.50% but this compound was cytotoxic to the tested C6 cells (cell viability: 11.83 ± 8.24%). Compounds **6**, **12**, and **21** were mild neurotrophic agents (120.09 ± 2.72%, 135.10 ± 5.59%, and 125.00 ± 1.50%, respectively) and the other compounds were inactive (<100%).

### 3.4. Cytotoxicity of the Isolated Compounds (**1**–**24**)

Podophyllotoxin is a representative anticancer natural product with a lignan scaffold and its synthetic derivative etoposide (VePesid^®^) is chemotherapy medication used for the treatments of diverse types of cancer [2]. Since most of the isolated phytochemicals (**1–24**) are derivatives of lignan or its monomer, phenylpropanoid, their cytotoxic activity against four human cancer cell lines, A549 (non-small-cell lung adenocarcinoma), SK-OV-3 (ovary malignant ascites), SK-MEL-2 (skin melanoma), and MKN-1 (adenosquamous carcinoma) were evaluated via the SRB bioassay method with etoposide as a positive control substance (IC_50_ 0.98, 2.15, 1.80, and 3.47 μM, respectively). As a result, four 8–*O*–4′ type neolignans, **1**−**4**, and a phenolic glucoside **24** showed an inhibitory effect on MKN-1 cancer cell with IC_50_ values of 11.15, 14.03, 14.16, 7.96, and 19.04 μM, respectively (Table 5).

## 4. Conclusions

A total of 24 phenolic compounds (**1**−**24**) including three new compounds (**1**, **9**, and **11**) were isolated and characterized from the twigs of *C. sinensis*. All isolates were evaluated for their anti-neuroinflammatory, neurotrophic, and cytotoxic activities. As a result, compounds **1**−**3**, 8–*O*–4′ type neolignans, displayed significant results in all activity evaluations. Compounds **6**, **12** and **21** showed anti-neuroinflammatory effects and/or neurotrophic activity. Compounds **24** displayed inhibitory effect on MKN-1 cancer cell. Through these results, it was suggested that the phenolic compounds in *C. sinensis* would be responsible for one of the various activities of this plant which could be the basis for the traditional use of *C. sinensis* for inflammatory diseases. Especially, 8–*O*–4′ type neolignans showed promising multi-target potentials as a drug against neurodegenerative diseases.

## Figures and Tables

**Table 1:** ^1^H (700 MHz) and ^13^C (175 MHz) NMR data of compounds **1**–**3**, and **9**.

Pos.	1 ^1^		2 ^1^		3 ^1^		9 ^2^	
	δ_C_	δ_H_ (multi, *J* in Hz)	δ_C_	δ_H_ (multi, *J* in Hz)	δ_C_	δ_H_ (multi, *J* in Hz)	δ_C_	δ_H_ (multi, *J* in Hz)
1	130.3				130.6		134.3	
2	102.7	6.58 (s)		6.97 (d, 1.6)	102.6	6.59 (s)	105.1	6.75 (s)
3	147.2				147.2		149.5	
4	134.2				134.0		136.4	
5	147.2			6.86 (d, 8.1)	147.2		149.5	
6	102.7	6.58 (s)		6.75 (dd, 8.1, 1.6)	102.6	6.59 (s)	105.1	6.75 (s)
7	73.1	4.99 (brs)	72.6	5.00 (d, 3.5)	72.7	4.99 (d, 3.2)	85.2	5.03 (d, 8.4)
8	87.5	4.23 (ddd, 6.6, 3.5, 3.0)	87.2	4.11 (dt, 6.7, 2.9)	87.2	4.10 (dt, 6.8, 3.1)	51.8	2.51 (m)
9a	60.7	3.93 (overlap)	60.8	3.87 (overlap)	60.8	3.87 (overlap)	67.8	3.82 (m)
9b		3.54 (m)		3.48 (overlap)		3.46 (m)		3.57 (dd, 9.9, 4.9)
1′	133.1		133.1		133.1		134.7	
2′/6′	105.6	7.29 (s)	105.5	6.48 (s)	105.5	6.49 (s)	104.8	6.76 (s)
3′/5′	153.6		153.3		153.3		149.5	
4′	139.9		138.8		138.9		136.3	
7′	199.1		34.4	2.70 (m)	34.4	2.71 (m)	84.5	5.06 (d, 8.4)
8′	40.4	3.23 (t, 5.4)	32.8	1.91 (m)	32.8	1.92 (m)	54.6	2.38 (m)
9′a	58.3	4.06 (t, 5.3)	62.3	3.71 (t, 6.3)	62.3	3.72 (t, 6.3)	61.6	3.78 (dd, 11.2, 5.2)
9′b								3.69 (dd, 11.2, 4.4)
1′′							102.3	4.70 (d, 1.3)
2′′							72.3	3.81 (m)
3′′							72.7	3.65 (dd, 4.5, 3.2)
4′′							74.1	3.40 (m)
5′′							70.3	3.61 (dq, 9.5, 6.5)
6′′							18.2	1.27 (d, 6.5)
3-OCH_3_	56.5	3.88 (s)	56.1	3.90 (s)	56.5	3.88 (s)	57.0	3.90 (s)
5-OCH_3_	56.5	3.88 (s)			56.5	3.88 (s)	57.0	3.90 (s)
3′,5′-OCH_3_	56.6	3.96 (s)	56.3	3.87 (s)	56.3	3.88 (s)	57.0	3.90 (s)
4-OH		5.47 (s)		5.55 (s)		5.45 (s)		

^1^ Measured in chloroform-*d*; ^2^ Measured in methanol-*d*_4_.

**Table 2:** ^1^H (700 MHz) and ^13^C (175 MHz) NMR data of compound **11** in methanol-*d*_4._

Pos.	1^1^	
	δ_C_	δ_H_ (multi, *J* in Hz)
1	134.6	
2/6	107.6	7.23 (s)
3/5	154.4	
4	140.7	
7	200.0	
8	42.1	3.11 (t, 6.1)
9	58.8	3.86 (t, 6.1)
1′	104.6	5.00 (d, 7.5)
2′	75.9	3.40 (dd, 8.8, 7.5)
3′	78.0	3.33 (t, 8.8)
4′	71.5	3.30 (t, 8.8)
5′	78.6	3.12 (ddd, 8.8, 5.3, 2.3)
6′a	62.7	3.67 (dd, 11.9, 2.3)
6′b		3.55 (dd, 11.9, 5.3)
3,5-OCH3	57.3	3.81 (s)

**Table 3:** Inhibitory effects of selected compounds on nitric oxide (NO) production in lipopolysaccharide (LPS)-activated BV-2 cells.

Compound	IC_50_ (μM) ^1^	Cell viability (%) ^2^
**1**	18.66	113.97 ± 1.36
**2**	19.75	105.99 ± 7.78
**3**	18.63	94.33 ± 3.84
**6**	12.69	101.33 ± 8.15
**9**	97.50	97.67 ± 3.20
**12**	72.50	114.97 ± 5.17
**17**	68.77	106.59 ± 1.27
**20**	41.67	103.50 ± 2.62
**21**	15.00	103.50 ± 2.62
**24**	70.08	94.46 ± 10.89
l-NMMA ^3^	21.35	104.56 ± 4.20

^1^ IC_50_ value of each compound was defined as the concentration (μM) that caused 50% inhibition of NO production in LPS-activated BV-2 cells. ^2^ Cell viability after treatment with 20 μM of each compound was determined by 3-[4,5-dimethylthiazol-2-yl]-2,5-diphenyltetrazolium bromide (MTT) assay and is expressed in percentage (%). The results are averages of three independent experiments, and the data are expressed as mean ± SD. ^3^ *N*^G^-monomethyl-l-arginine (l-NMMA) as positive control.

**Table 4:** Effects of selected compounds on nerve growth factor (NGF) secretion in C6 cells.

Compound	NGF Secretion ^1^	Cell Viability (%) ^2^
**1**	110.27 ± 2.18	96.59 ± 2.78
**2**	127.98 ± 3.95	101.75 ± 2.40
**3**	149.89 ± 1.97	105.00 ± 3.21
**6**	120.09 ± 2.72	109.25 ± 8.03
**12**	135.10 ± 5.59	94.41 ± 1.83
**20**	157.73 ± 1.50	11.83 ± 8.24
**21**	125.00 ± 1.50	117.97 ± 8.50
6-shogaol ^3^	149.53 ± 5.36	97.00 ± 0.17

^1^ C6 cells were treated with 20 μM of compounds. After 24 h, the content of NGF secretion in C6-conditioned media was measured by ELISA. The level of secreted NGF cells is expressed as percentage of the untreated control. The data shown represent the means ± SD of three independent experiments performed in triplicate. ^2^ Cell viability after treatment with 20 μM of each compound was determined by MTT assay and is expressed in percentage (%). The results are averages of three independent experiments, and the data are expressed as mean ± SD. ^3^ 6-shogaol as positive control.

**Table 5:** Cytotoxicity of selected compounds against four cultured human cell lines.

Compound	IC_50_ (μM) ^1^			
	A-549	SK-OV-3	SK-MEL-2	MKN-1
**1**	>30.0	>30.0	>30.0	11.15
**2**	>30.0	>30.0	>30.0	14.03
**3**	>30.0	>30.0	>30.0	14.16
**4**	>30.0	>30.0	>30.0	7.96
**24**	>30.0	>30.0	>30.0	19.04
Etoposide ^2^	0.98	2.15	1.80	3.47

^1^ 50% inhibitory concentration; the concentration of compound that caused a 50% inhibition in cell growth. ^2^ Etoposide as positive control.

## Data Availability

The data presented in this study are available on request from the corresponding author.

## Supplementary Materials

The following are available online at https://www.mdpi.com/article/10.3390/antiox10040551/s1, General Experimental Procedures: Detailed isolation process. Scheme S1. Fractionation and isolation process of compounds **1**–**24** from *C. sinensis* twigs. Figures S1–S7: HRMS, NMR, and ECD spectra of **1**, Figures S8–S14: HRMS, NMR, and ECD spectra of **9**. Figure S15: Extracted ion chromatograms (EICs, *m*/*z* 431.1311) of chiral derivatized l- and d-rhamnopyranose purchased or obtained by hydrolysis of **9**. Figures S16–S21: HRMS and NMR spectra of **11**. Figure S22: Extracted ion chromatograms (EICs, *m*/*z* 447.1260) of chiral derivatized d- and l-glucopyranose purchased or obtained by hydrolysis of **11**. Figures S23–S26: ^1^H and ^13^C NMR spectra of **2** and **3**.
