# Supplementary material for: Phenolic Constituents of Chinese Quince Twigs (Chaenomeles sinensis Koehne) and Their Anti-Neuroinflammatory, Neurotrophic, and Cytotoxic Activities
Source: Antioxidants (Basel). 2021 Apr 2;10(4):551. doi: 10.3390/antiox10040551 (PMC8067024; doi:10.3390/antiox10040551)

Phenolic constituents of Chinese Quince (*Chaenomeles sinensis* Koehne) and their anti-neuroinflammatory, neurotrophic, and cytotoxic activities

Dong Hyun Kim,[†] Lalita Subedi,[‡] Hye Ryeong Kim,[†] Sang Un Choi,[§] Sun Yeou Kim,[‡,⊥] Chung Sub Kim[*,†,∇]

[†]School of Pharmacy, Sungkyunkwan University, Suwon 16419, Republic of Korea

[‡]Gachon Institute of Pharmaceutical Science, Gachon University, Incheon 21936, Republic of Korea

[§]Korea Research Institute of Chemical Technology, Daejeon 34114, Republic of Korea

[⊥]College of Pharmacy, Gachon University, #191, Hambakmoero, Yeonsu-gu, Incheon 21936, Republic of Korea

[∇]Department of Biopharmaceutical Convergence, Sungkyunkwan University, Suwon, 16419, Republic of Korea

Corresponding Authors:

*Tel: +82-31-290-7727. E-mail: chungsub.kim@skku.edu

Contents

General Experimental Procedures. (Detailed isolation process) 1

Scheme S1. Fractionation and isolation process of compounds **1**–**24** from *C. sinensis* twigs. 2

Figure S1. HRESIMS spectrum of **1** 0

Figure S2. ECD spectrum of **1** 1

Figure S3. ^{1}H NMR spectrum of **1** in chloroform-*d* (700 MHz) 2

Figure S4. ^{13}C NMR spectrum of **1** in chloroform-*d* (175 MHz) 3

Figure S5. ^{1}H-^{1}H COSY spectrum of **1** in chloroform-*d* 4

Figure S6. HSQC spectrum of **1** in chloroform-*d* 5

Figure S7. HMBC spectrum of **1** in chloroform-*d* 6

Figure S8. HRFABMS spectrum of **9** 7

Figure S9. ECD spectra of **9** (top) and **25** (bottom, adopted from the original paper). 8

Figure S10. ^{1}H NMR spectrum of **9** in methanol-d_4 (700 MHz) 9

Figure S11. ^{13}C NMR spectrum of **9** in methanol-d_4 (175 MHz) 10

Figure S12. ^{1}H-^{1}H COSY spectrum of **9** in methanol-d_4 11

Figure S13. HSQC spectrum of **9** in methanol-d_4 12

Figure S14. HMBC spectrum of **9** in methanol-d_4 13

Figure S15. Extracted ion chromatograms (EICs, *m*/*z* 431.1311) of chiral derivatized L- and D-rhamnopyranose purchased or obtained by hydrolysis of **9**. 14

Figure S16. HRESIMS spectrum of **11** 15

Figure S17. ^{1}H NMR spectrum of **11** in methanol-d_4 (700 MHz) 16

Figure S18. ^{13}C NMR spectrum of **11** in methanol-d_4 (175 MHz) 17

Figure S19. ^{1}H-^{1}H COSY spectrum of **11** in methanol-d_4 18

Figure S20. HSQC spectrum of **11** in methanol-d_4 19

Figure S21. HMBC spectrum of **11** in methanol-d_4 20

Figure S22. Extracted ion chromatograms (EICs, *m*/*z* 447.1260) of chiral derivatized D- and L-glucopyranose purchased or obtained by hydrolysis of **11**. 21

Figure S23. ^{1}H NMR spectrum of **2** in chloroform-*d* (700 MHz) 22

Figure S24. ^{13}C NMR spectrum of **2** in chloroform-*d* (700 MHz) 23

Figure S25. ^{1}H NMR spectrum of **3** in chloroform-*d* (700 MHz) 24

Figure S26. ^{13}C NMR spectrum of **3** in chloroform-*d* (700 MHz) 25

General Experimental Procedures. (Detailed isolation process)

The $CHCl_3$ soluble layer (15 g) was applied to silica gel column chromatography ($CHCl_3$-MeOH, 50:1 → 1:1) to yield 12 fractions (C1-C12). Fraction C3 (600 mg) was subjected to RP-C_{18} silica gel column (60% aqueous MeOH) to give ten subfractions (C3a-C3j). Compound **6** (4 mg) was obtained by purification of fraction C3j using semipreparative HPLC (85% aqueous MeCN). Fraction C5 (1.1 g) separated with RP-C_{18} silica gel column with solvent system 70% aqueous MeOH to afford 12 subfractions (C5a-C5l). Subfraction C5a was fractionated over LiChroprep Lobar-A RP-C_{18} column with 35% aqueous MeCN and further purified by semipreparative HPLC (20% aqueous MeCN) to yield compounds **1** (2 mg), **2** (2 mg), **3** (2 mg) and **13** (2 mg). The EtOAc soluble layer (3 g) was subjected to a Sephadex LH-20 column (90% aqueous MeOH) to give five fractions (E1-E5). Fraction E1 (3.8 g) was applied to silica gel column chromatography ($CHCl_3$-MeOH, 10:1) to yield 15 subfractions (E1a-E1o). E1h (400 mg) was chromatographed over Lichroprep Lobar-A RP-C_{18} (60% aqueous MeOH) to give five subfractions (E1h1-E1h5). E1h1 was fractionated over Lichroprep Lopbar-A RP-C_{18} column (50% aqueous MeOH) followed by semipreparative HPLC (65% aqueous MeCN) to yield compound **24** (2 mg). Subfraction E1j (260 mg) was subjected to LiChroprep Lobar-A RP-C_{18} column with 40% aqueous MeOH to give five subfractions (E1j1-E1j5). Subfraction E1j1 was purified by semipreparative HPLC (15% aqueous MeCN) to yield compounds **20** (2 mg) and **21** (3 mg). *n*-BuOH soluble layer was applied to silica gel column chromatography eluted with $CHCl_3$-MeOH-H_2O (3:1:0.1) yielding 10 fractions (B1-B10). Fraction B3 (60 mg) was separated with RP-C_{18} Sep-pak (45% aqueous MeOH) and further purified by semipreparative HPLC (35% MeOH) to yield compound **7** (2 mg). Fraction B4 (1.9 g) was fractionated by RP-C18 silica gel column (40% aqueous MeOH) to give three subfractions (B4a-B4c). Subfraction B4a (1.5 g) was futher purified by semipreparative HPLC (35% aqueous MeCN) to yield compound **18** (70 mg). Further isolation of fraction B5 (500 mg) by RP-C18 silica gel column using 40% aqueous MeOH, yielded nine subfractions (B5a-B5i). Subfraction B5a was purified by semipreparative HPLC (15% aqueous MeCN) to obtain compounds **12** (5 mg) and **17** (17 mg). Subfraction B5c was purified by semipreparative HPLC (17% aqueous MeCN) to yield compounds **5** (2 mg), **9** (2 mg) and **10** (2 mg). Subfraction B5d was purified by semipreparative HPLC (17% aqueous MeCN) to give compounds **4** (5 mg) and **8** (3 mg). Subfraction B5i was purified by semipreparative HPLC (50% aqueous MeOH) to yield compounds **14** (3 mg) and **16** (4 mg). Fraction B6 (700 mg) was subjected to a RP-C_{18} silica gel chromatography eluted with 40% aqueous MeOH to give eight subfractions (B6a-B6h). Subfraction B6a (150 mg) was purified by semipreparative HPLC (7% aqueous MeCN) to obtain compounds **22** (4 mg). Subfraction B6c (120 mg) was purified by semipreparative HPLC with solvent system 25% aqueous MeOH to give compounds **19** (34 mg). Subfraction B6d (100 mg) was chromatographed over Lichroprep Lobar-A (EtOAc-MeOH-H_2O, 5:1:0.1) and further purified by semipreparative HPLC (25% aqueous MeOH) to obtain compounds **11** (5 mg). Subfraction B6e (40 mg) was purified by semipreparative HPLC with solvent system 30% aqueous MeOH to yield compounds **23** (3 mg). Subfraction B6h was purified by semipreparative HPLC (40% aqueous MeOH) to give compounds **15** (7 mg).

Scheme S1. Fractionation and isolation process of compounds **1**–**24** from *C. sinensis* twigs.

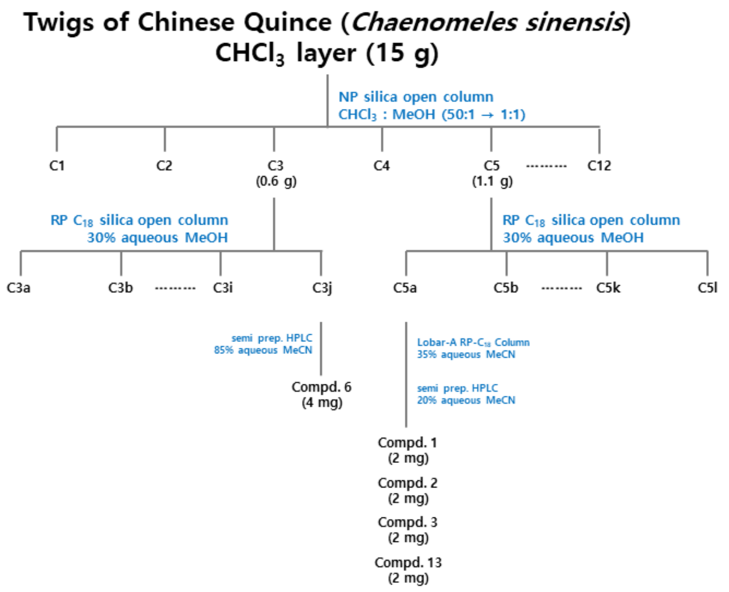

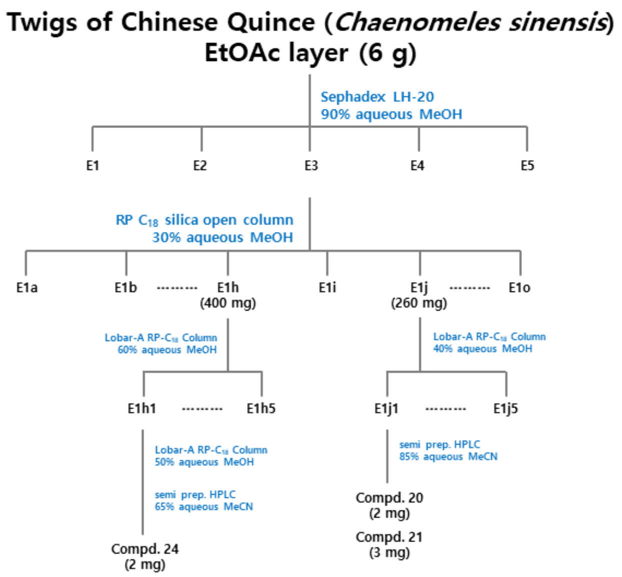

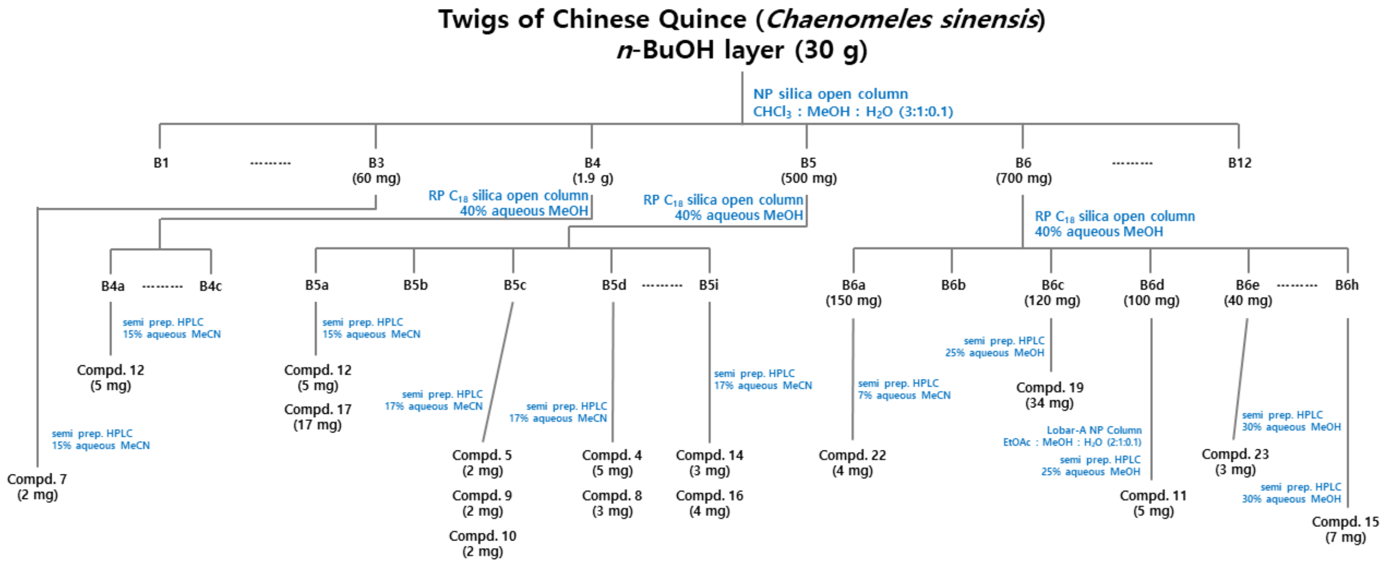

Figure S1. HRESIMS spectrum of **1**

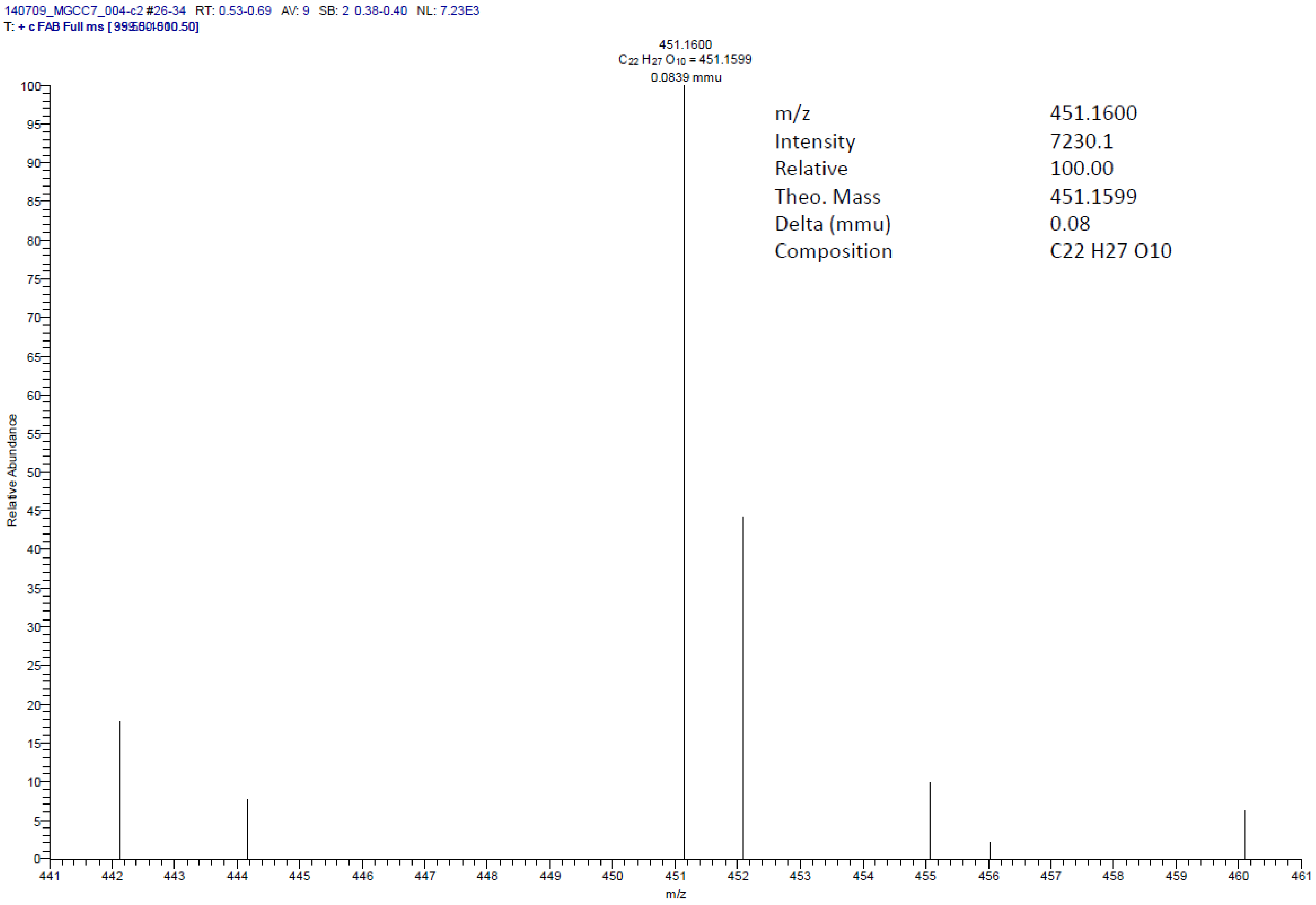

Figure S2. ECD spectrum of **1**

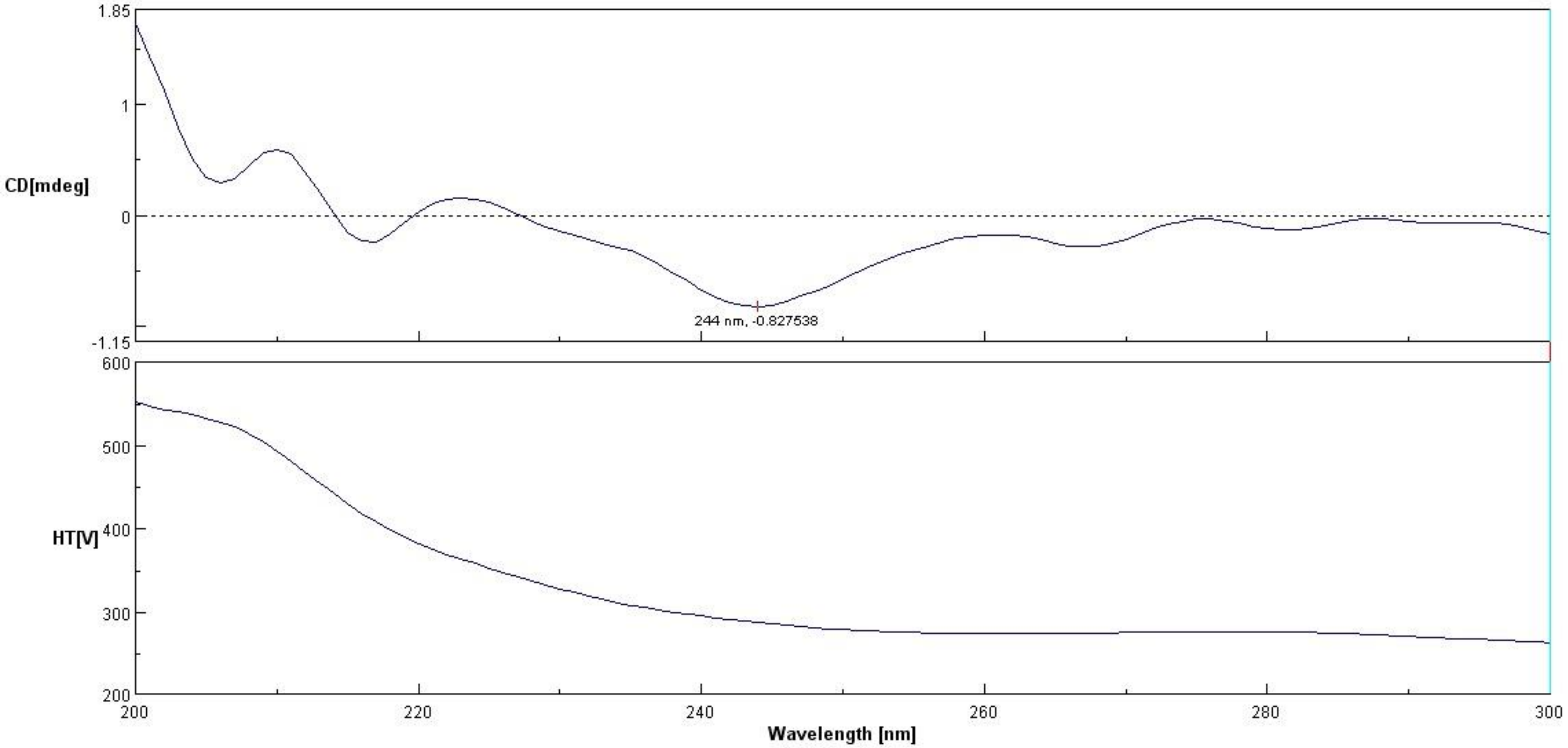

Figure S3. ^{1}H NMR spectrum of **1** in chloroform-*d* (700 MHz)

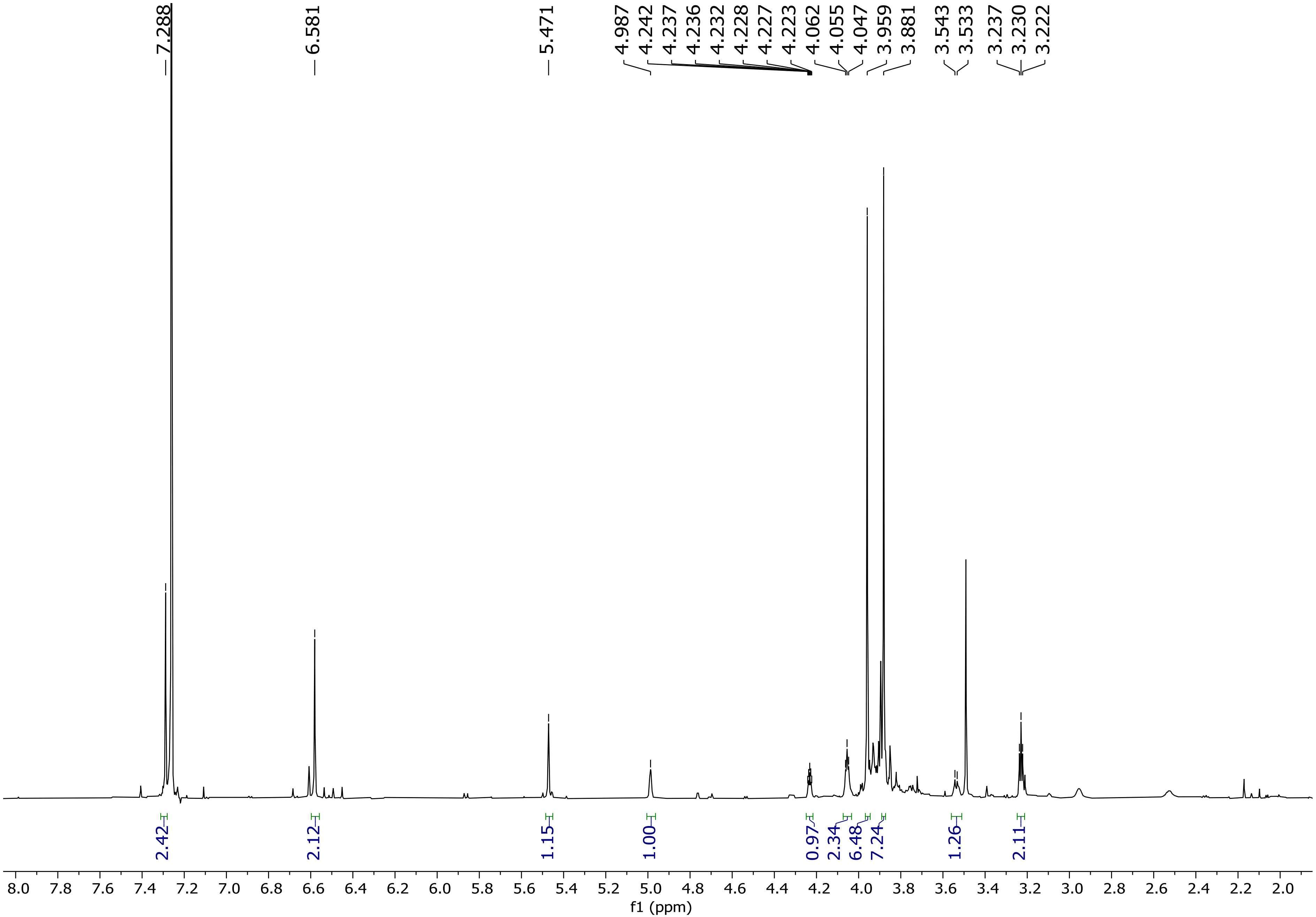

Figure S4. ^{13}C NMR spectrum of **1** in chloroform-*d* (175 MHz)

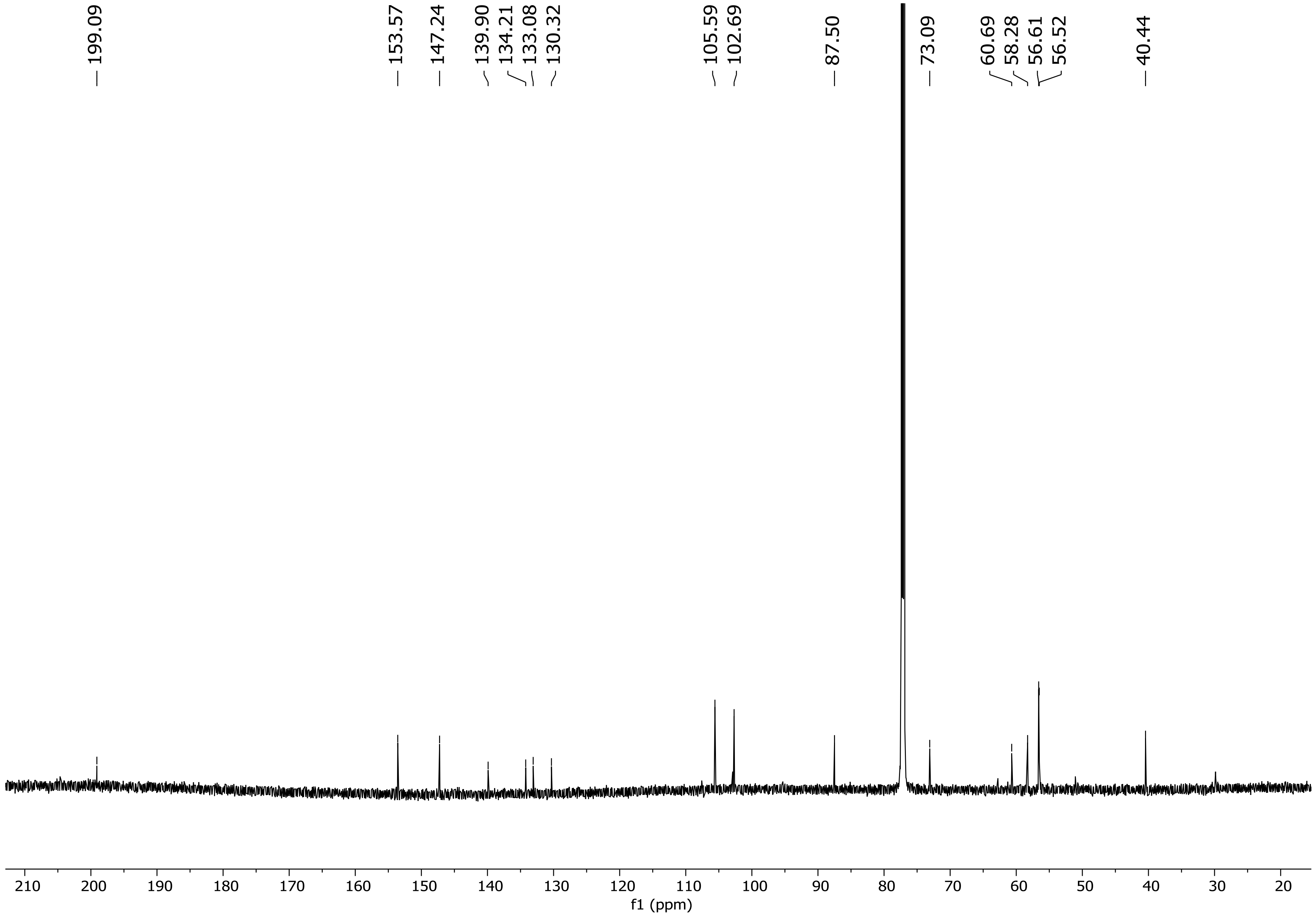

Figure S5. ^{1}H-^{1}H COSY spectrum of **1** in chloroform-*d*

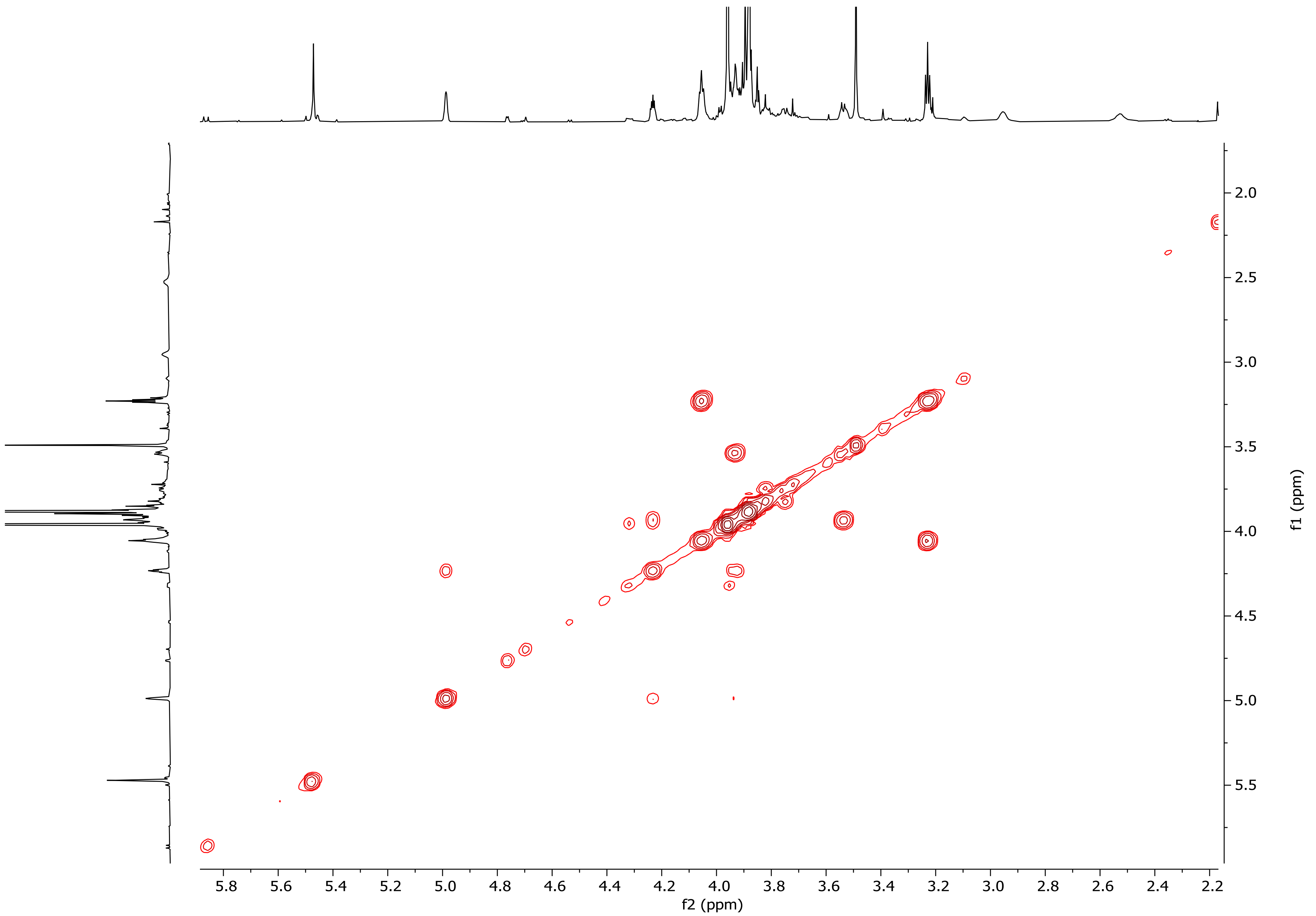

Figure S6. HSQC spectrum of **1** in chloroform-*d*

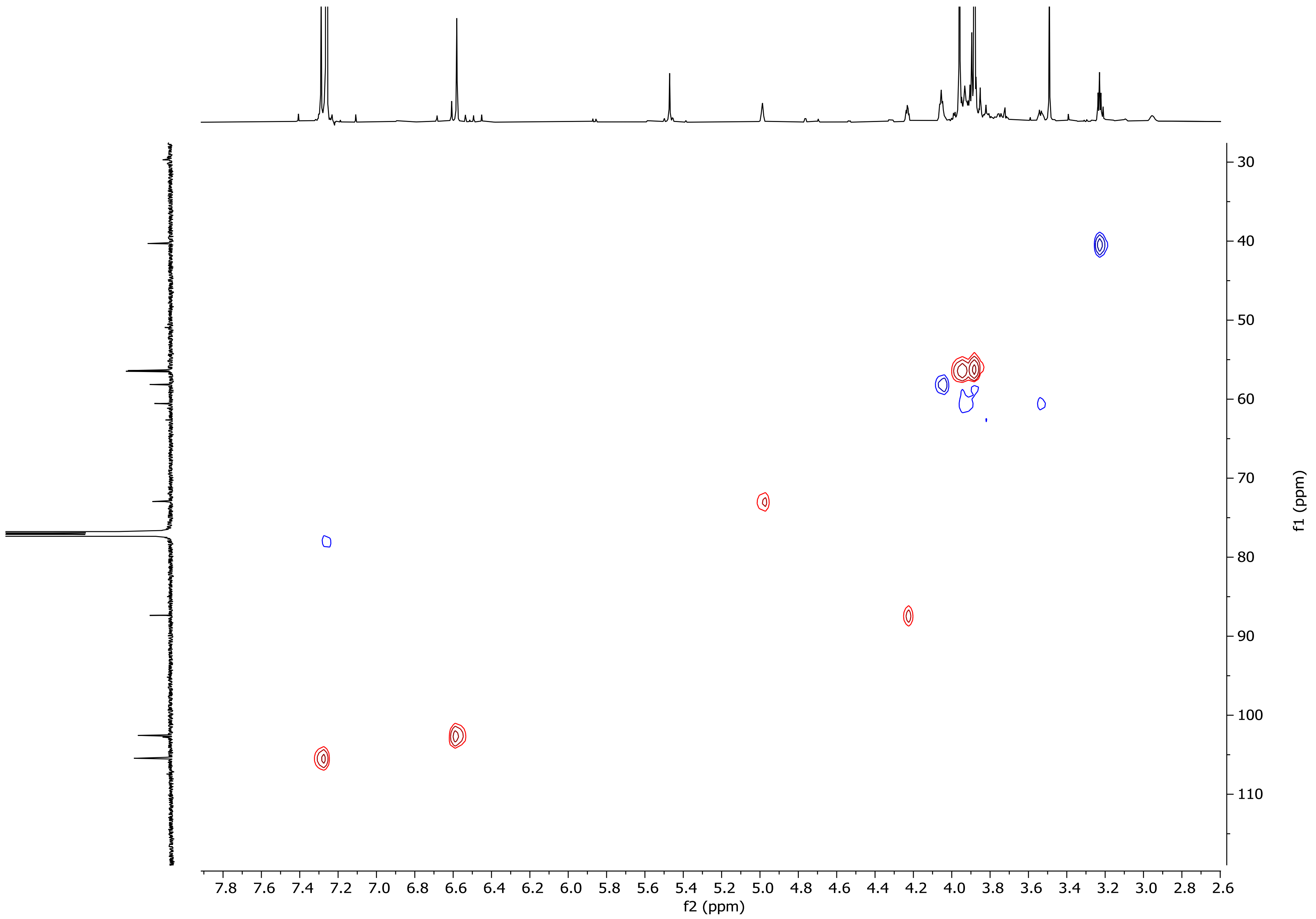

Figure S7. HMBC spectrum of **1** in chloroform-*d*

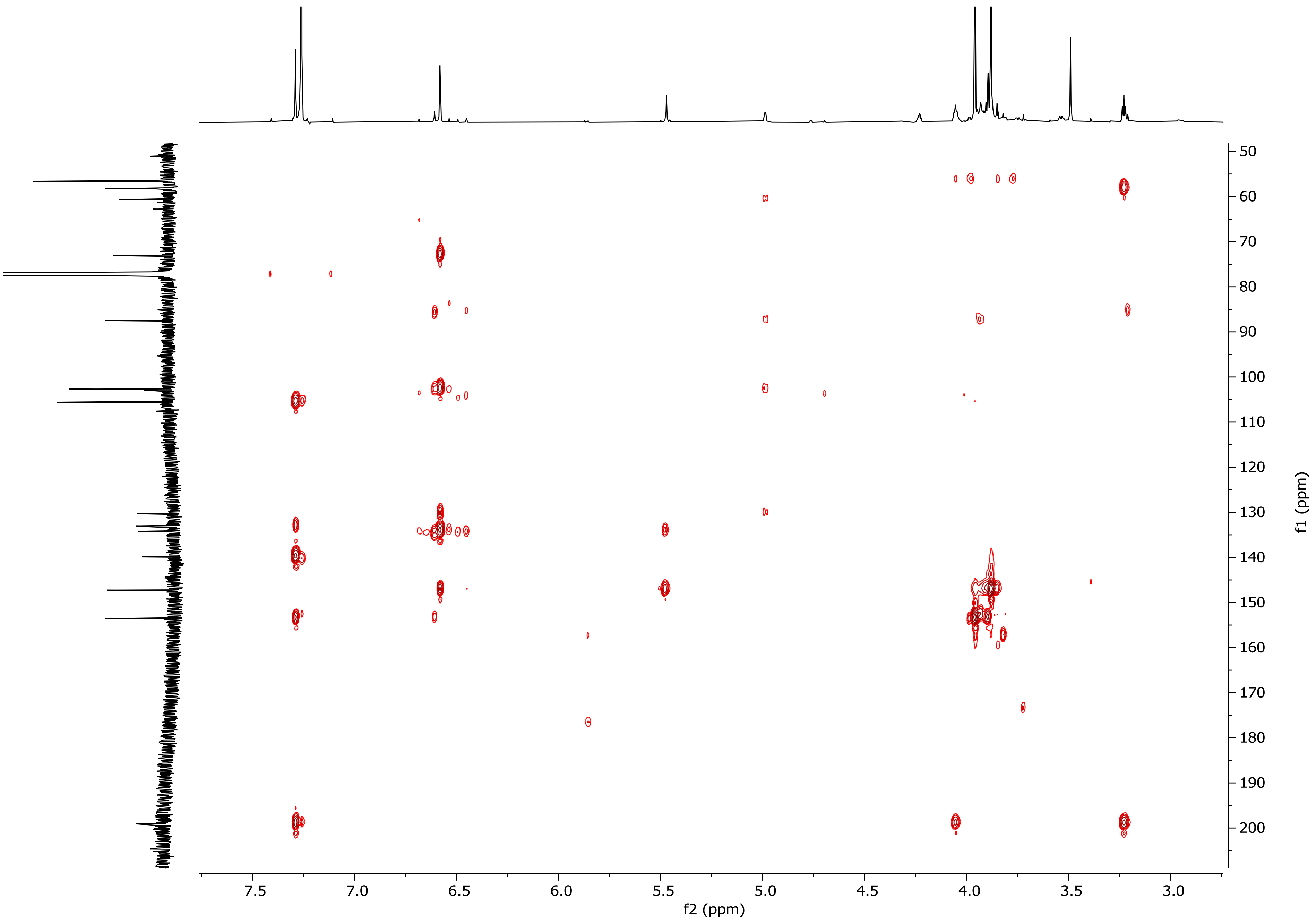

Figure S8. HRFABMS spectrum of **9**

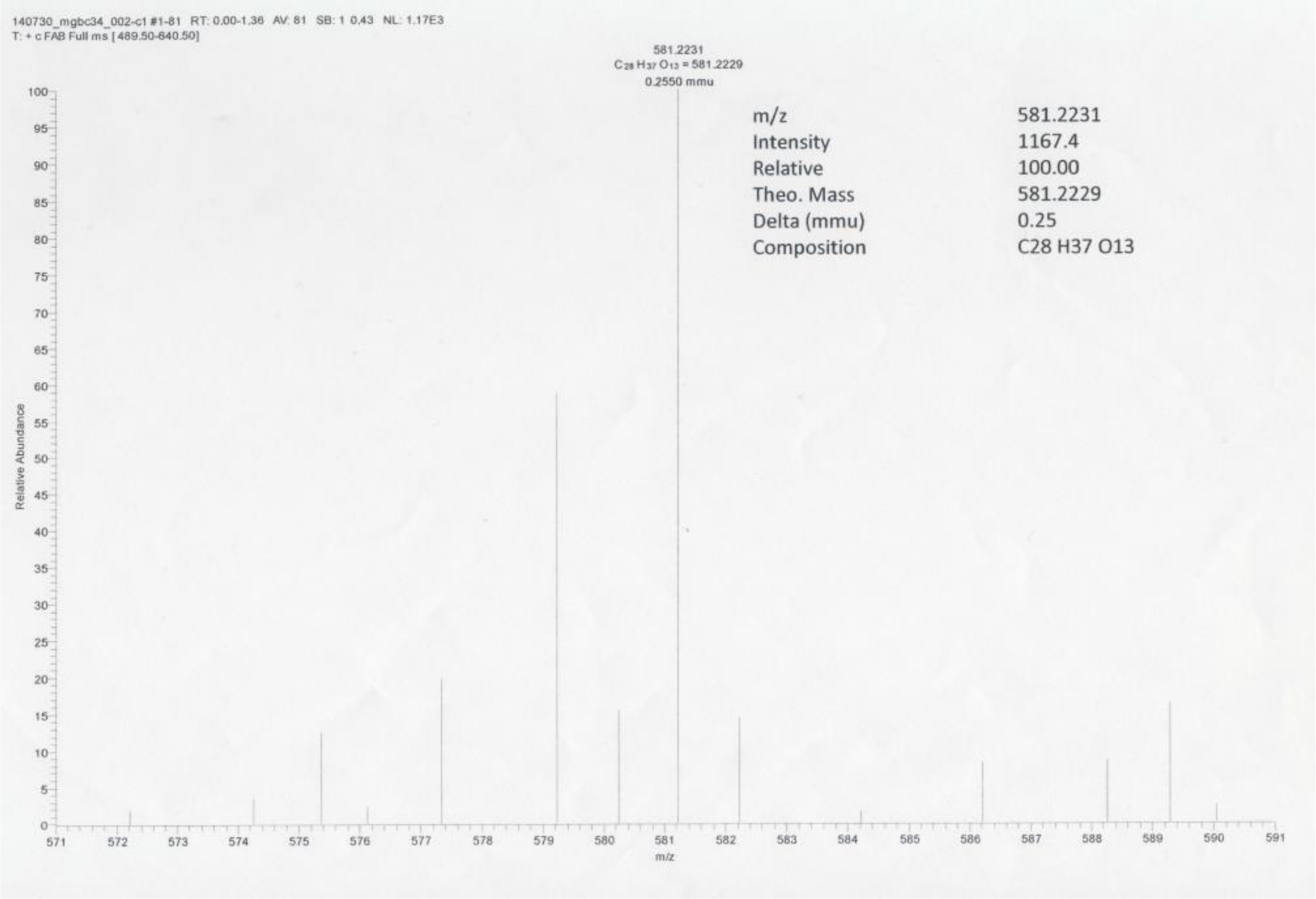

Figure S9. ECD spectra of **9** (top) and **25** (bottom, adopted from the original paper).

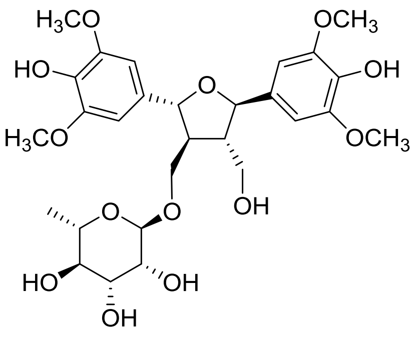

9

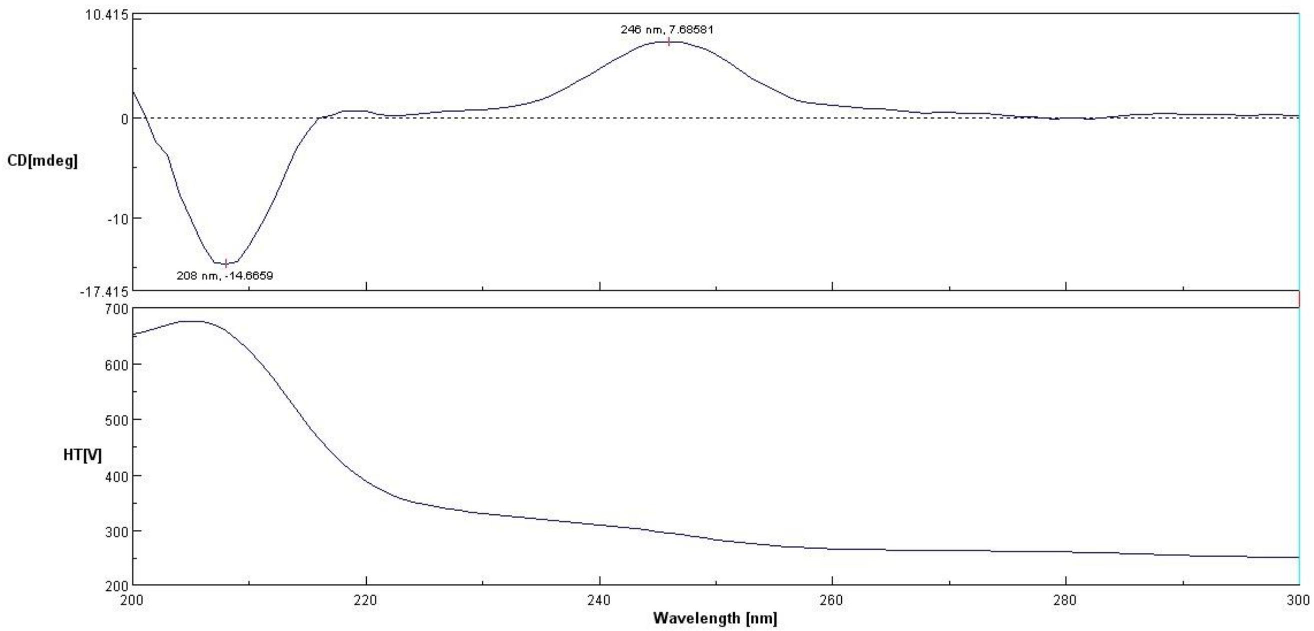

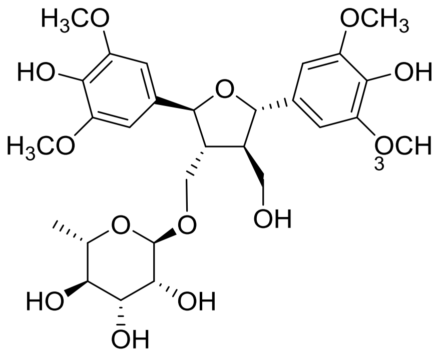

25

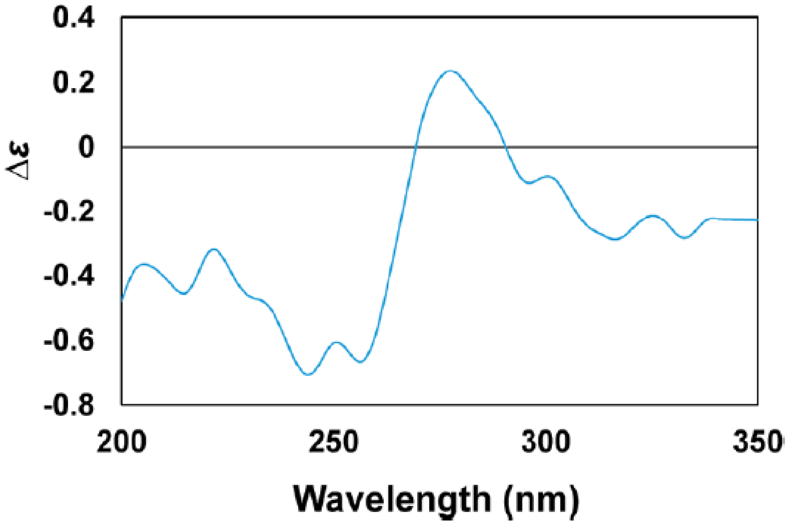

Figure S10. ^{1}H NMR spectrum of **9** in methanol-d_4 (700 MHz)

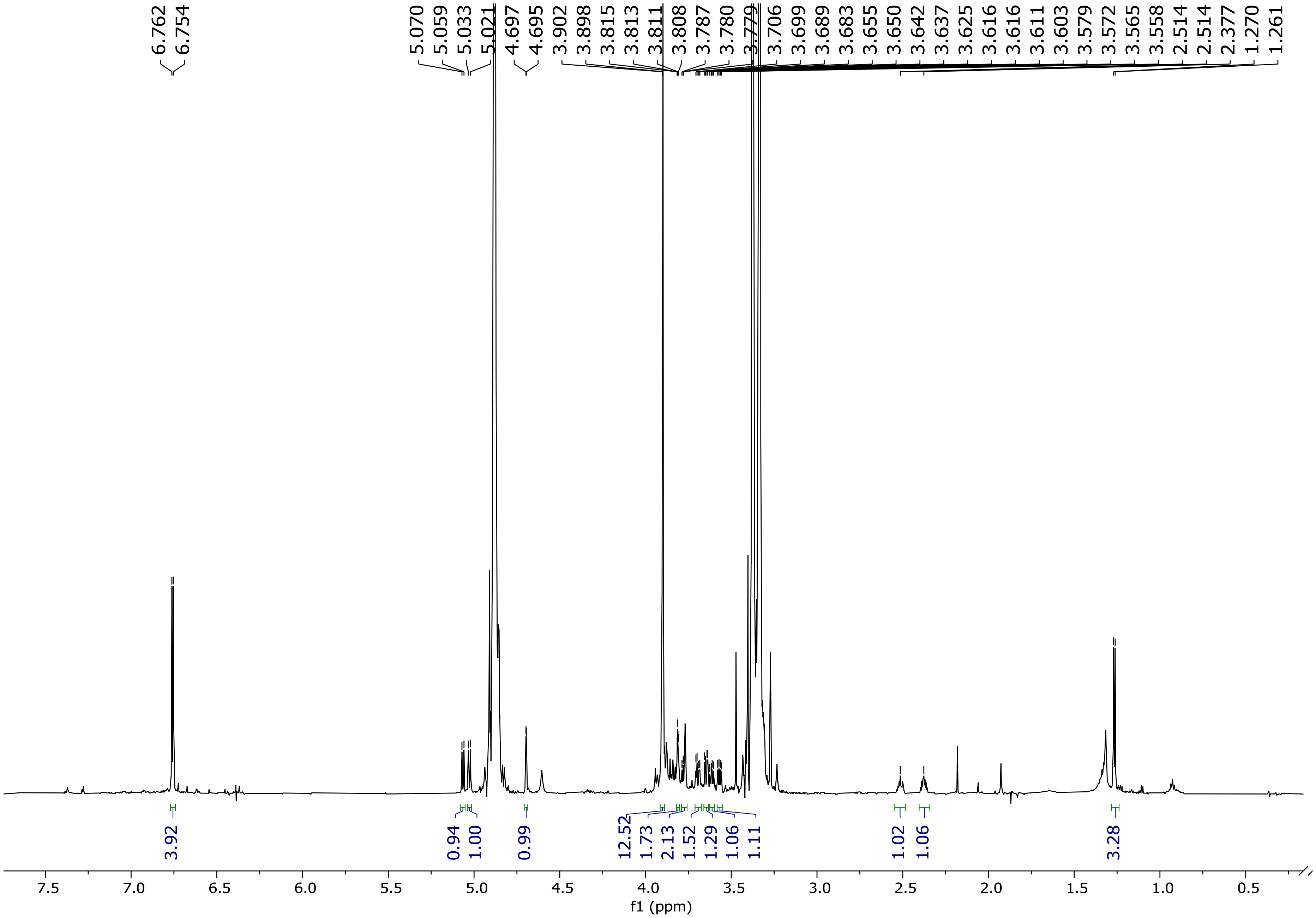

Figure S11. ^{13}C NMR spectrum of **9** in methanol-d_4 (175 MHz)

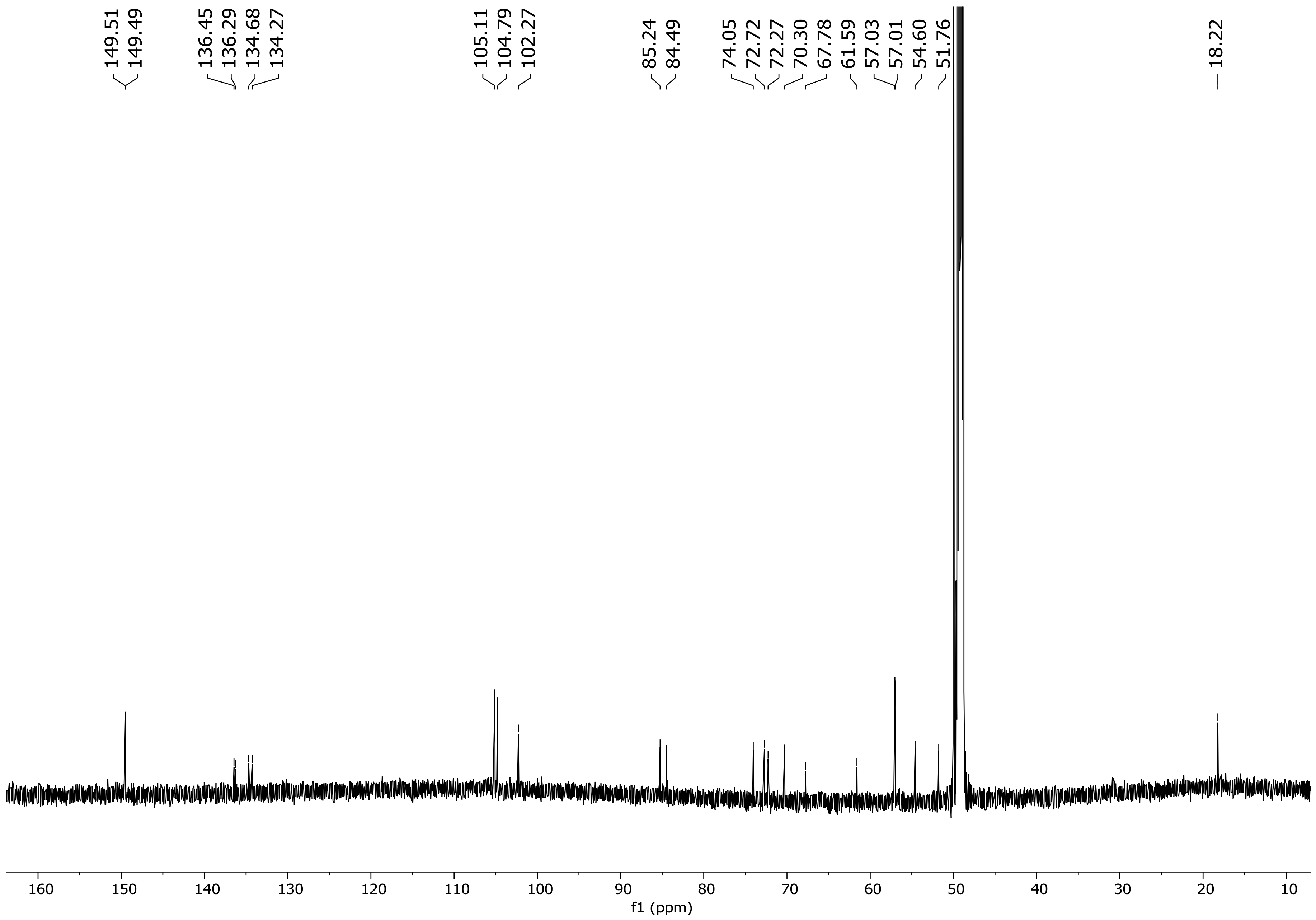

Figure S12. ^{1}H-^{1}H COSY spectrum of **9** in methanol-d_4

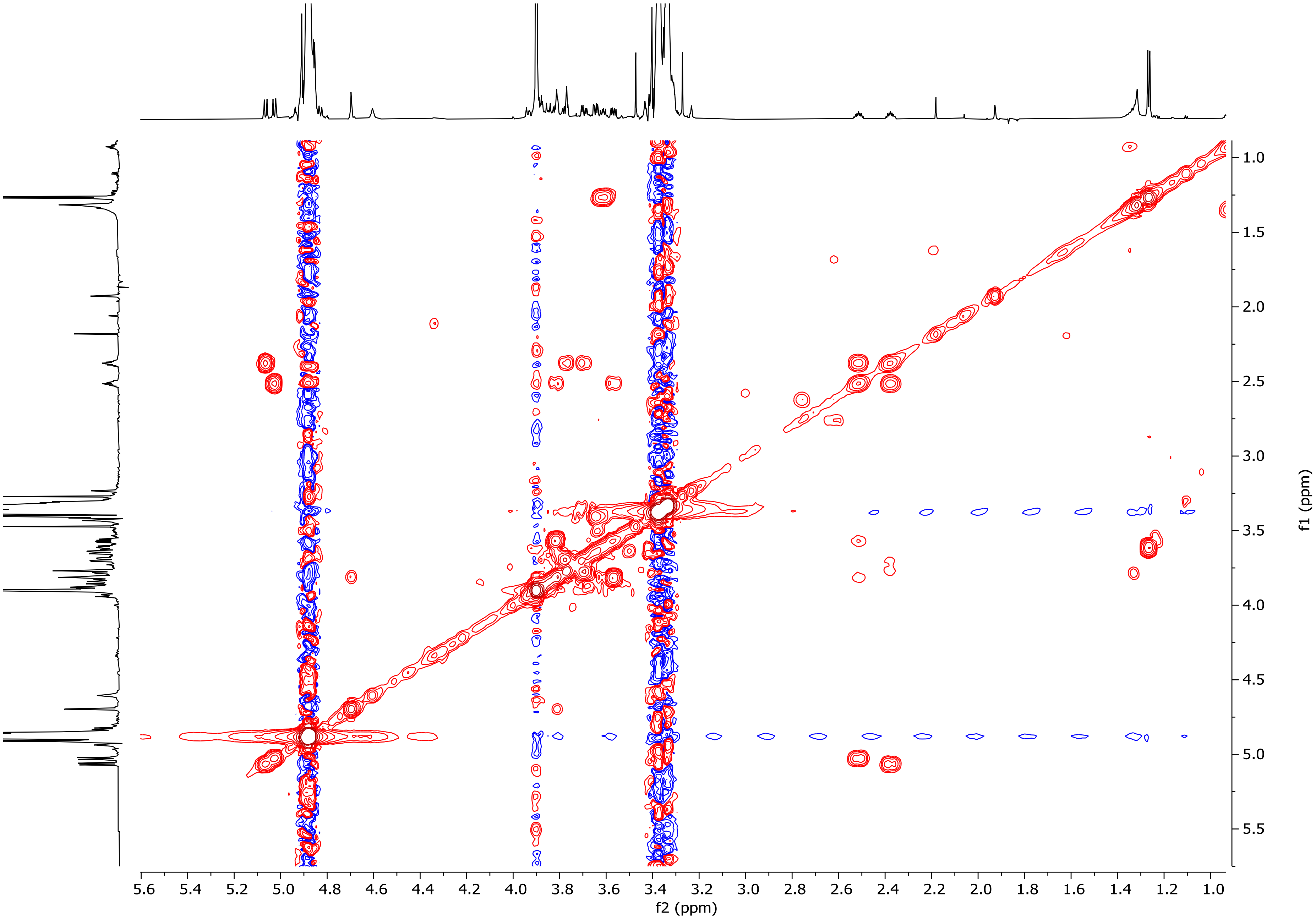

Figure S13. HSQC spectrum of **9** in methanol-d_4

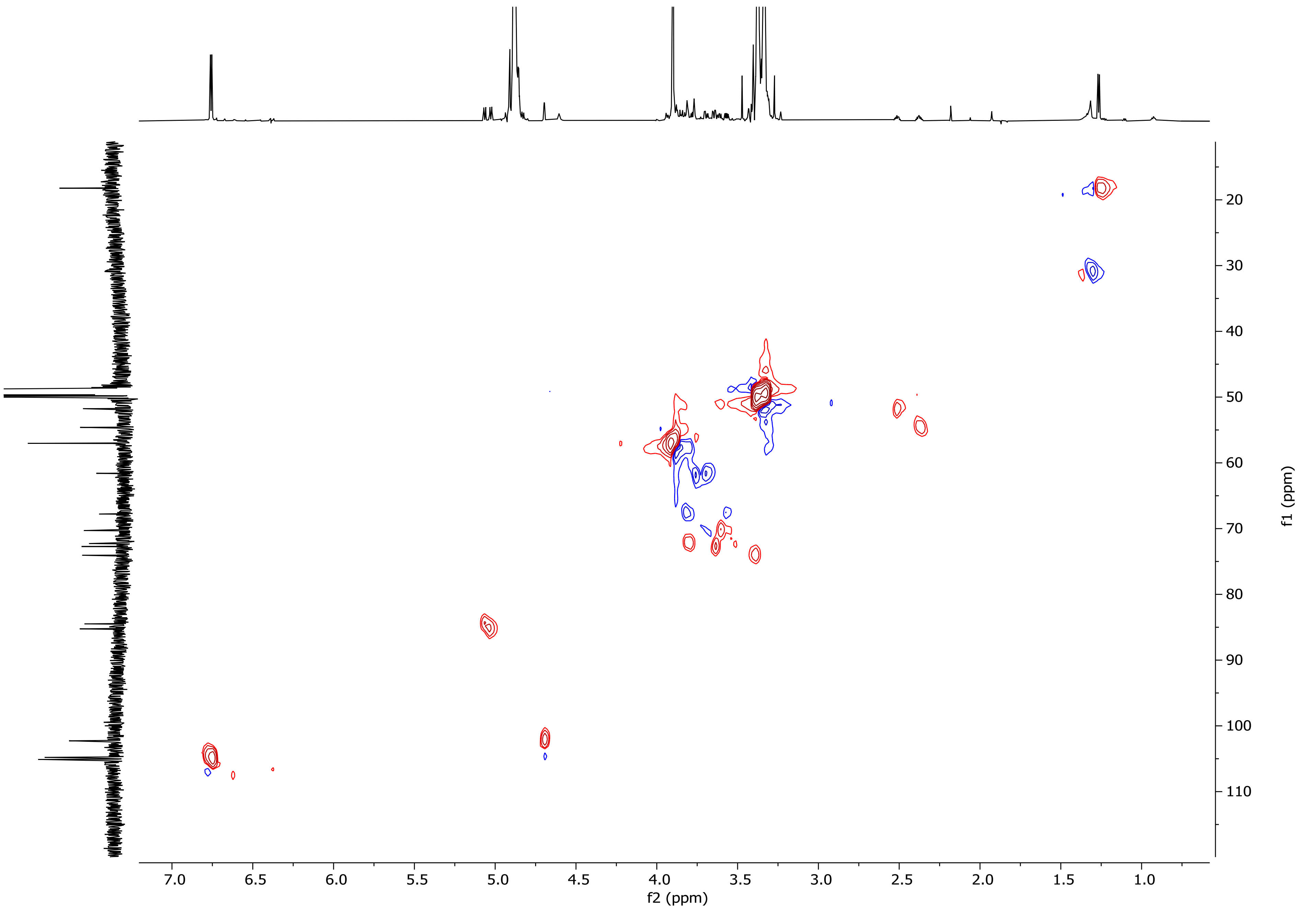

Figure S14. HMBC spectrum of **9** in methanol-d_4

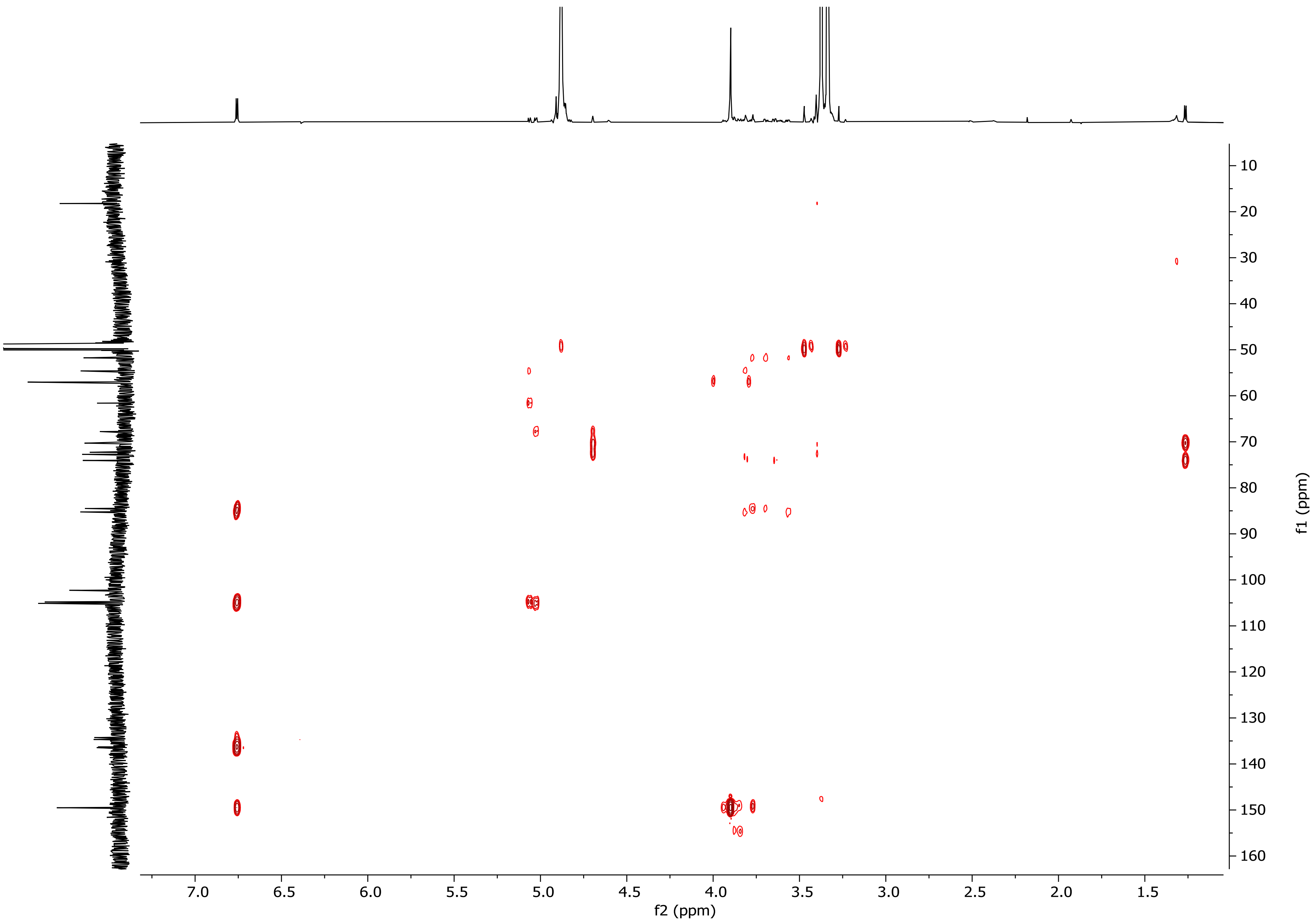

Figure S15. Extracted ion chromatograms (EICs, *m*/*z* 431.1311) of chiral derivatized L- and D-rhamnopyranose purchased or obtained by hydrolysis of **9**.

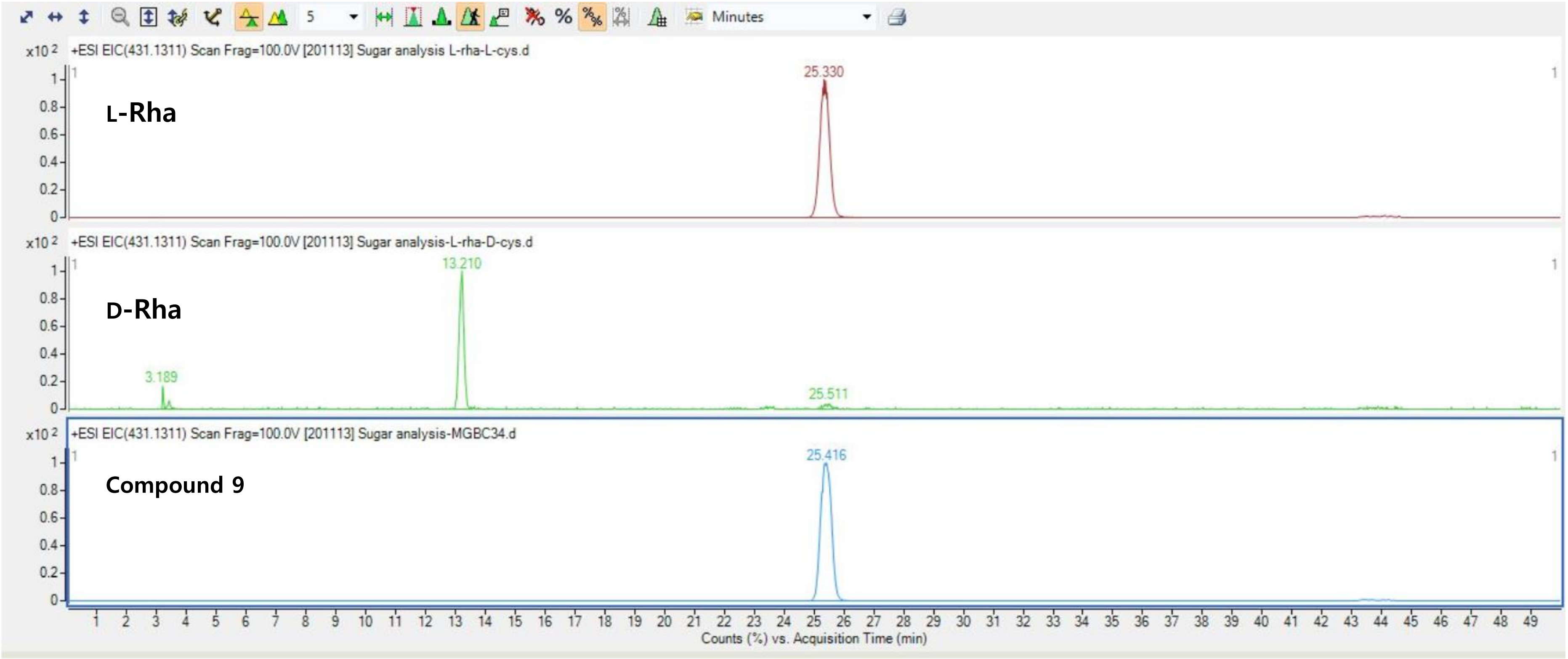

Figure S16. HRESIMS spectrum of **11**

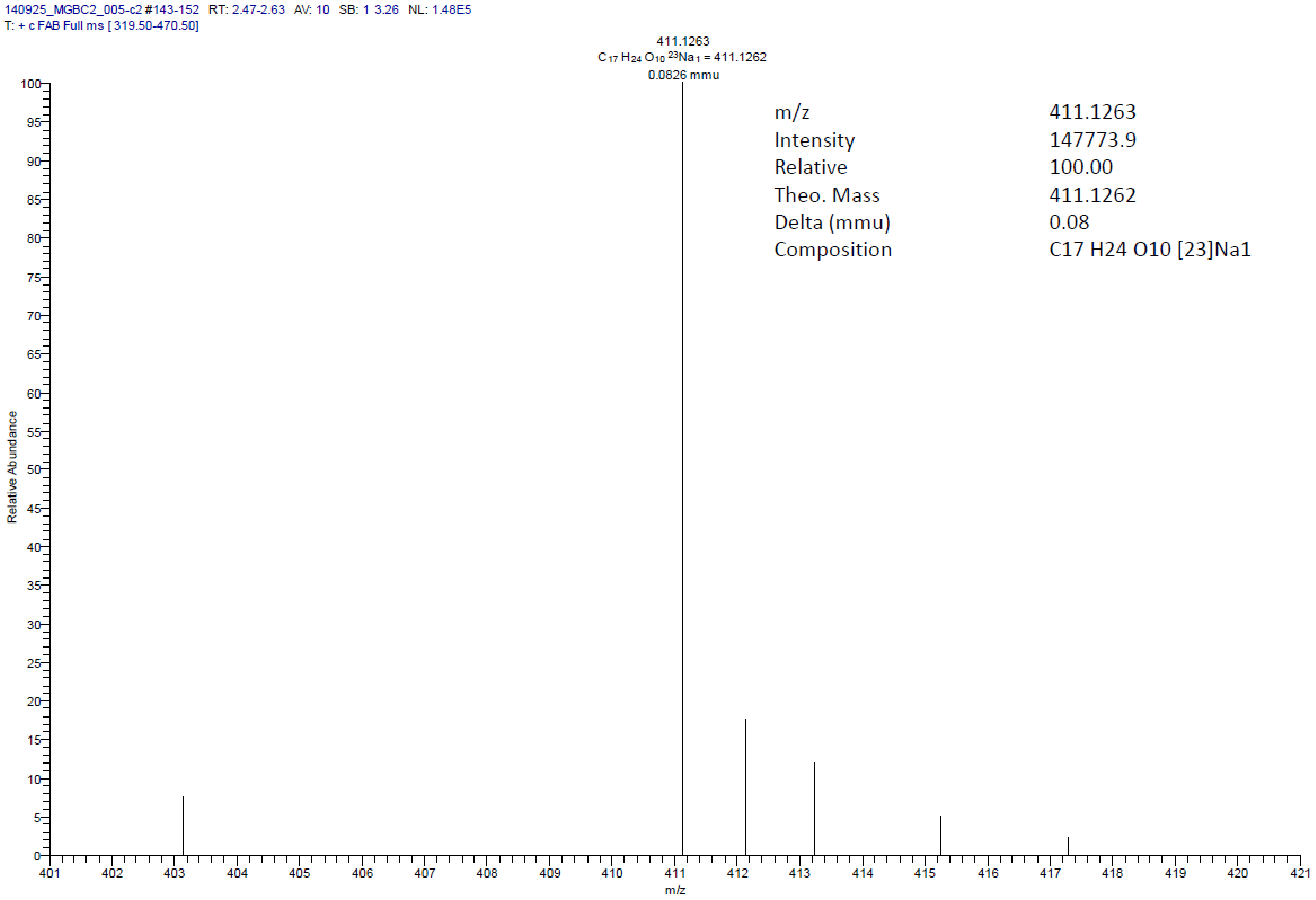

Figure S17. ^{1}H NMR spectrum of **11** in methanol-d_4 (700 MHz)

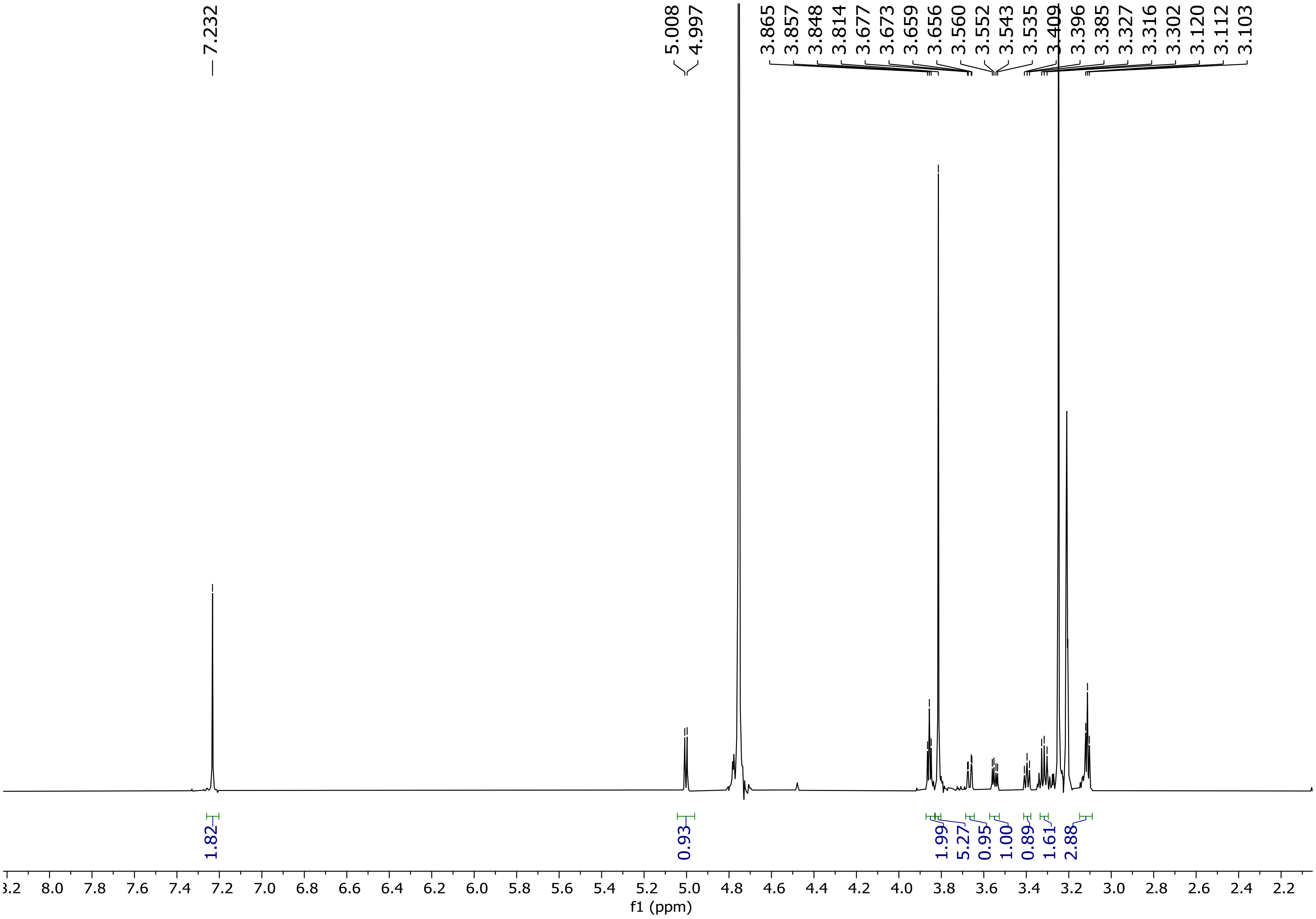

Figure S18. ^{13}C NMR spectrum of **11** in methanol-d_4 (175 MHz)

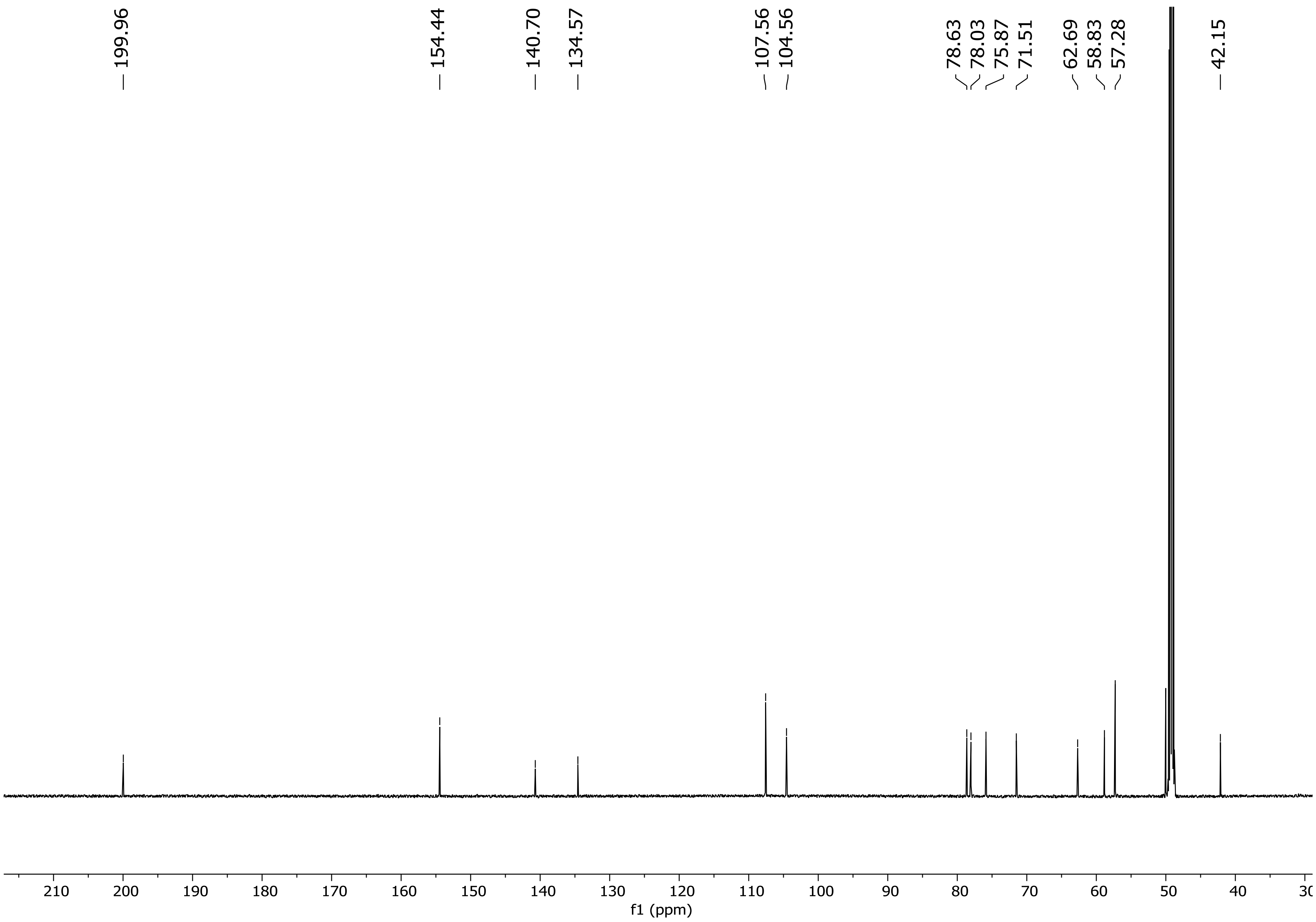

Figure S19. ^{1}H-^{1}H COSY spectrum of **11** in methanol-d_4

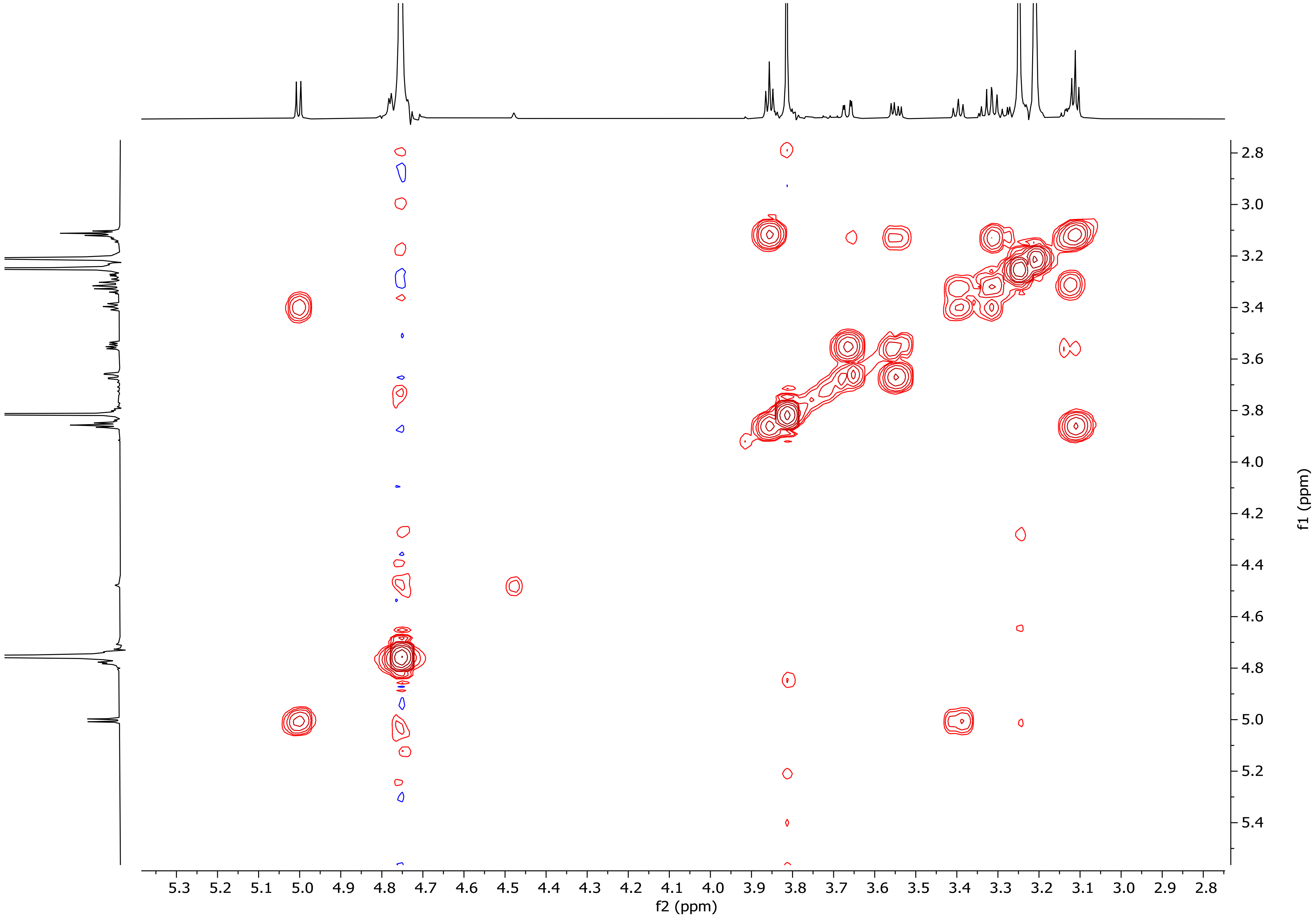

Figure S20. HSQC spectrum of **11** in methanol-d_4

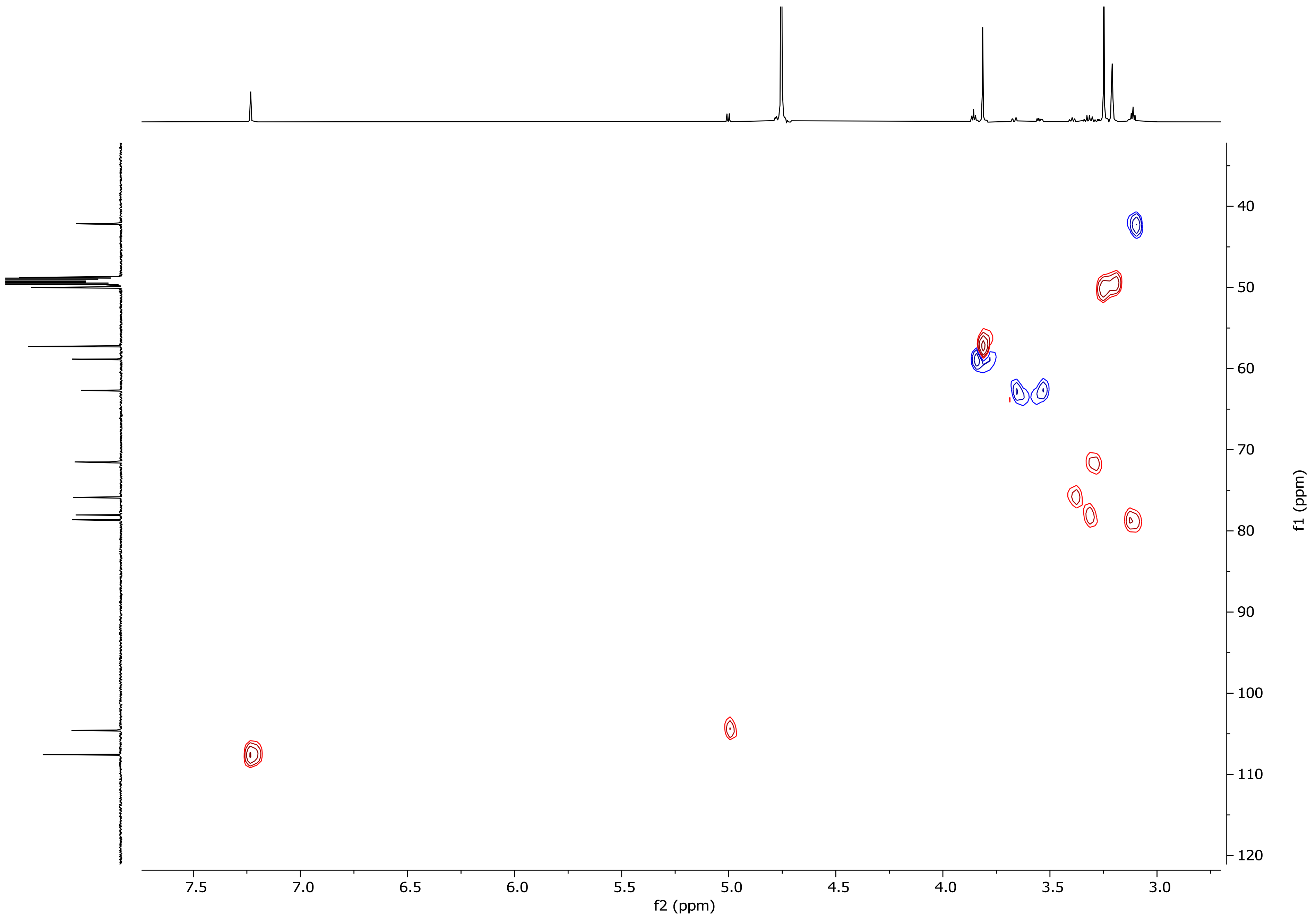

Figure S21. HMBC spectrum of **11** in methanol-d_4

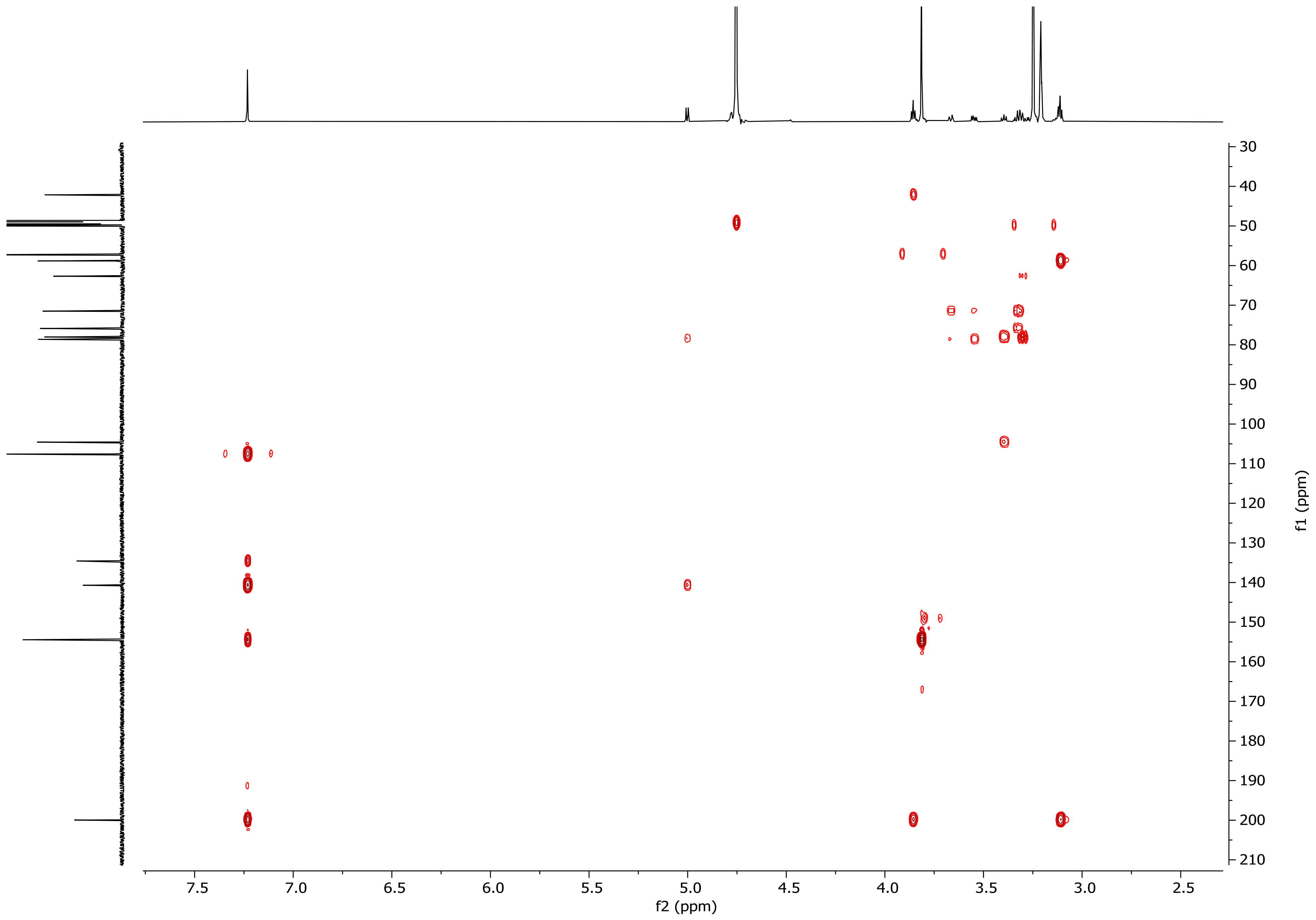

Figure S22. Extracted ion chromatograms (EICs, *m*/*z* 447.1260) of chiral derivatized D- and L-glucopyranose purchased or obtained by hydrolysis of **11**.

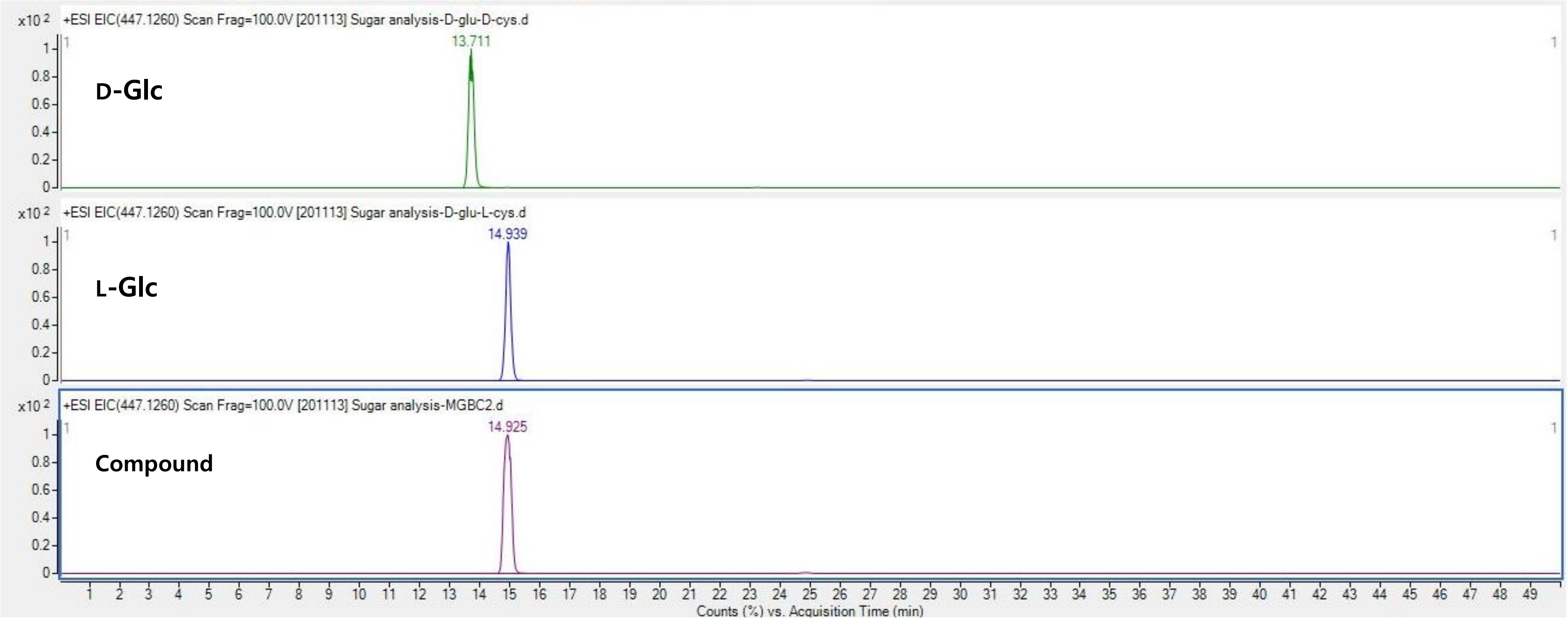

Figure S23. ^{1}H NMR spectrum of **2** in chloroform-d (700 MHz)

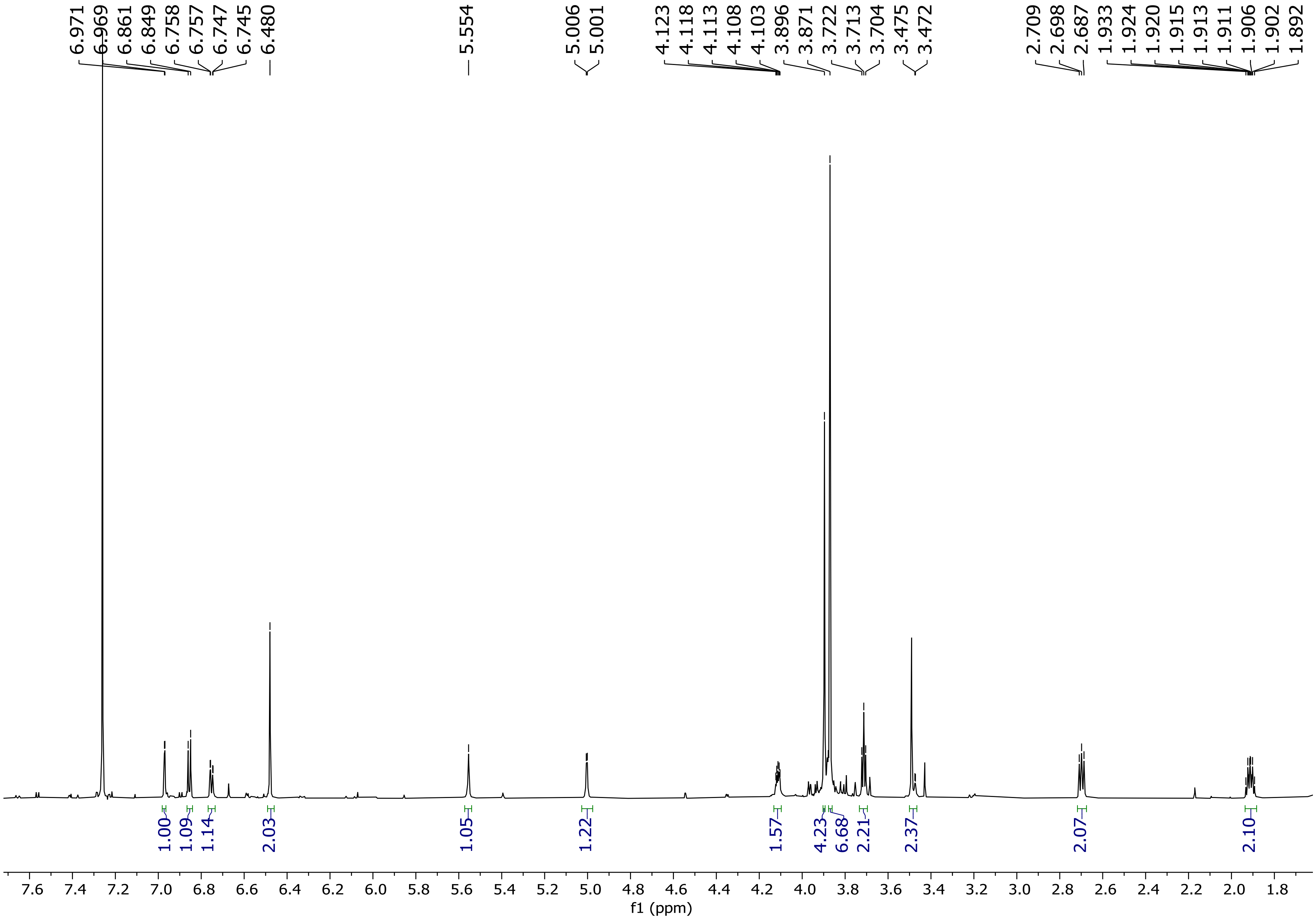

Figure S24. ^{13}C NMR spectrum of **2** in chloroform-*d* (700 MHz)

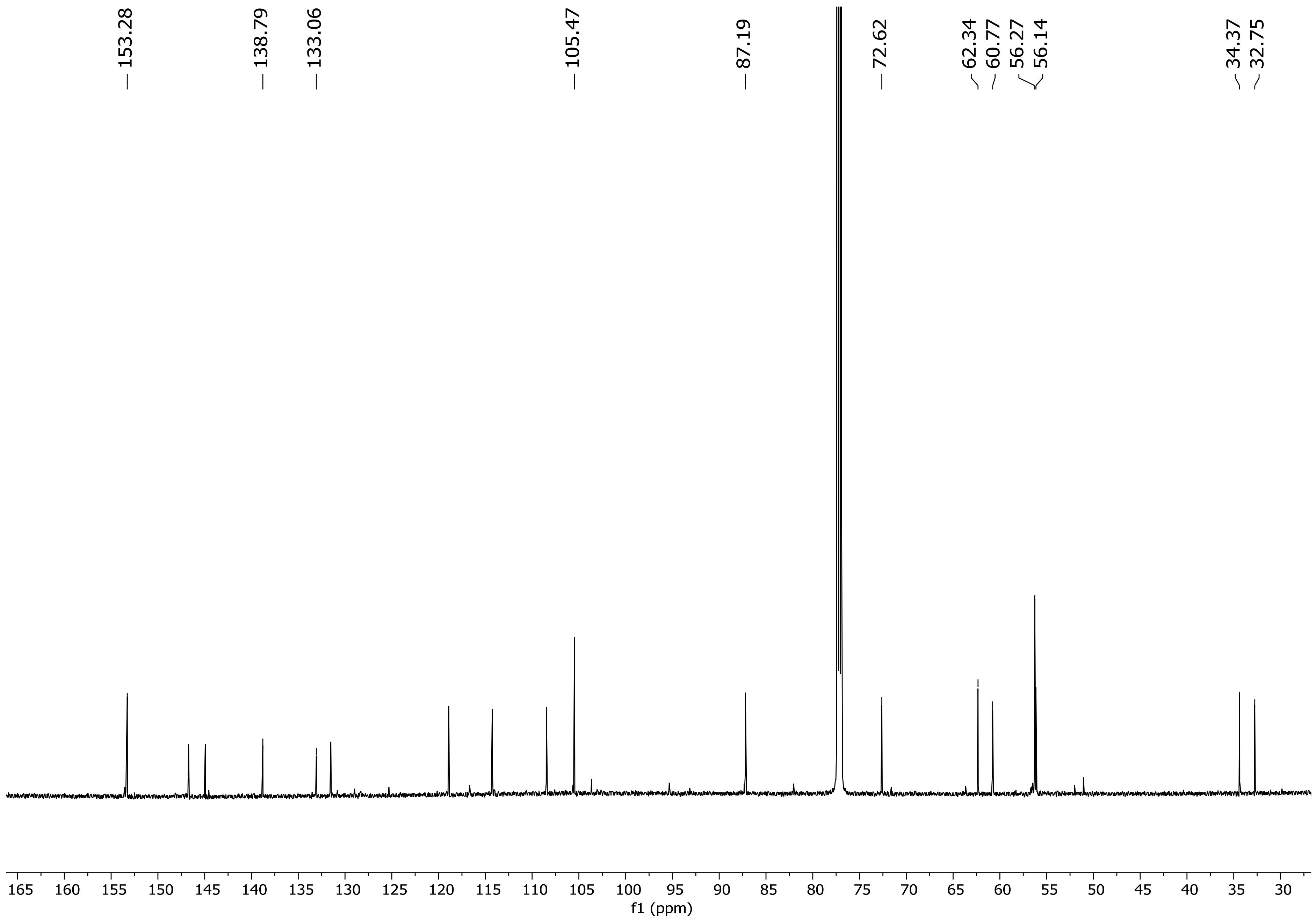

Figure S25. ^{1}H NMR spectrum of **3** in chloroform-*d* (700 MHz)

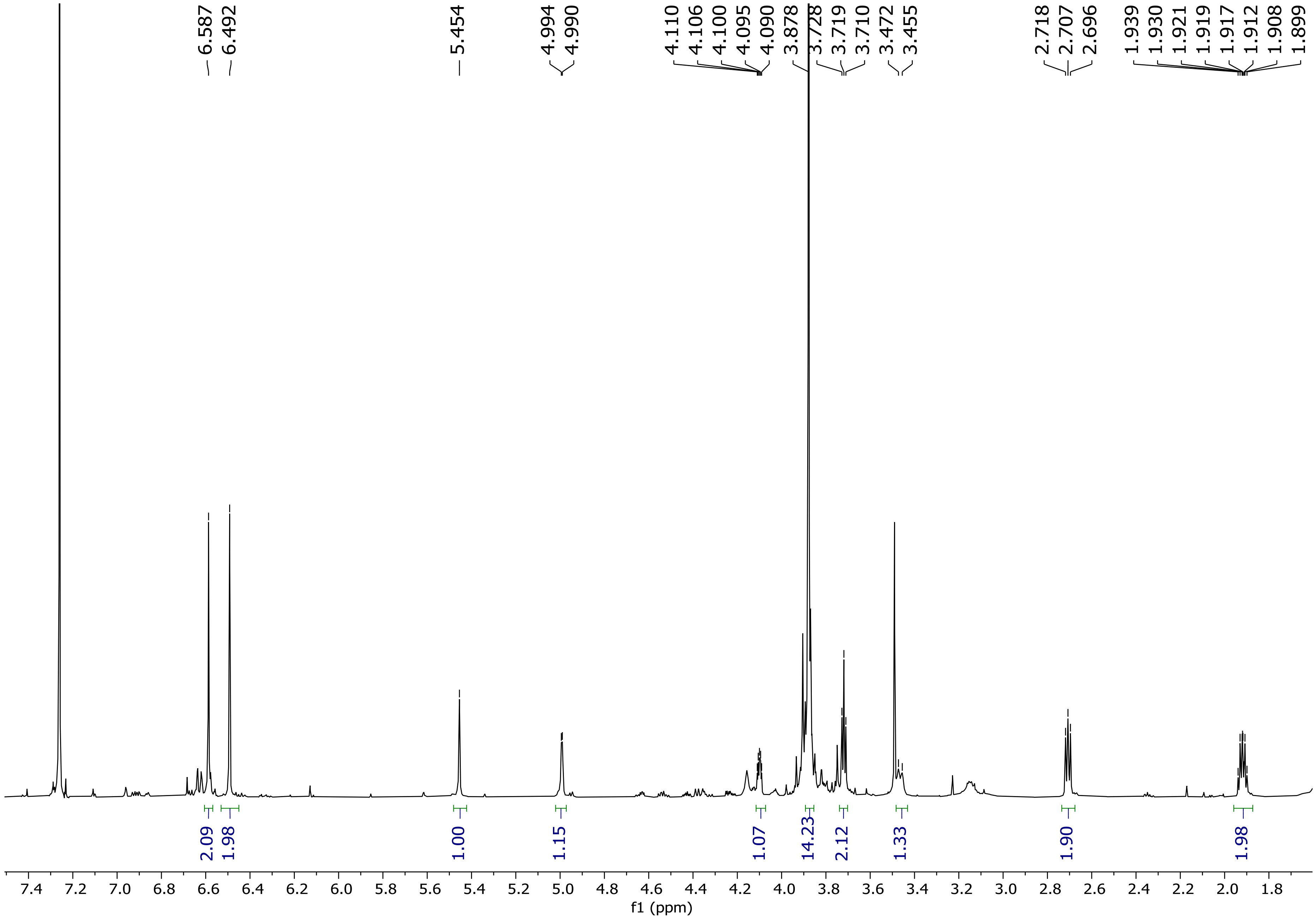

Figure S26. ^{13}C NMR spectrum of **3** in chloroform-*d* (700 MHz)

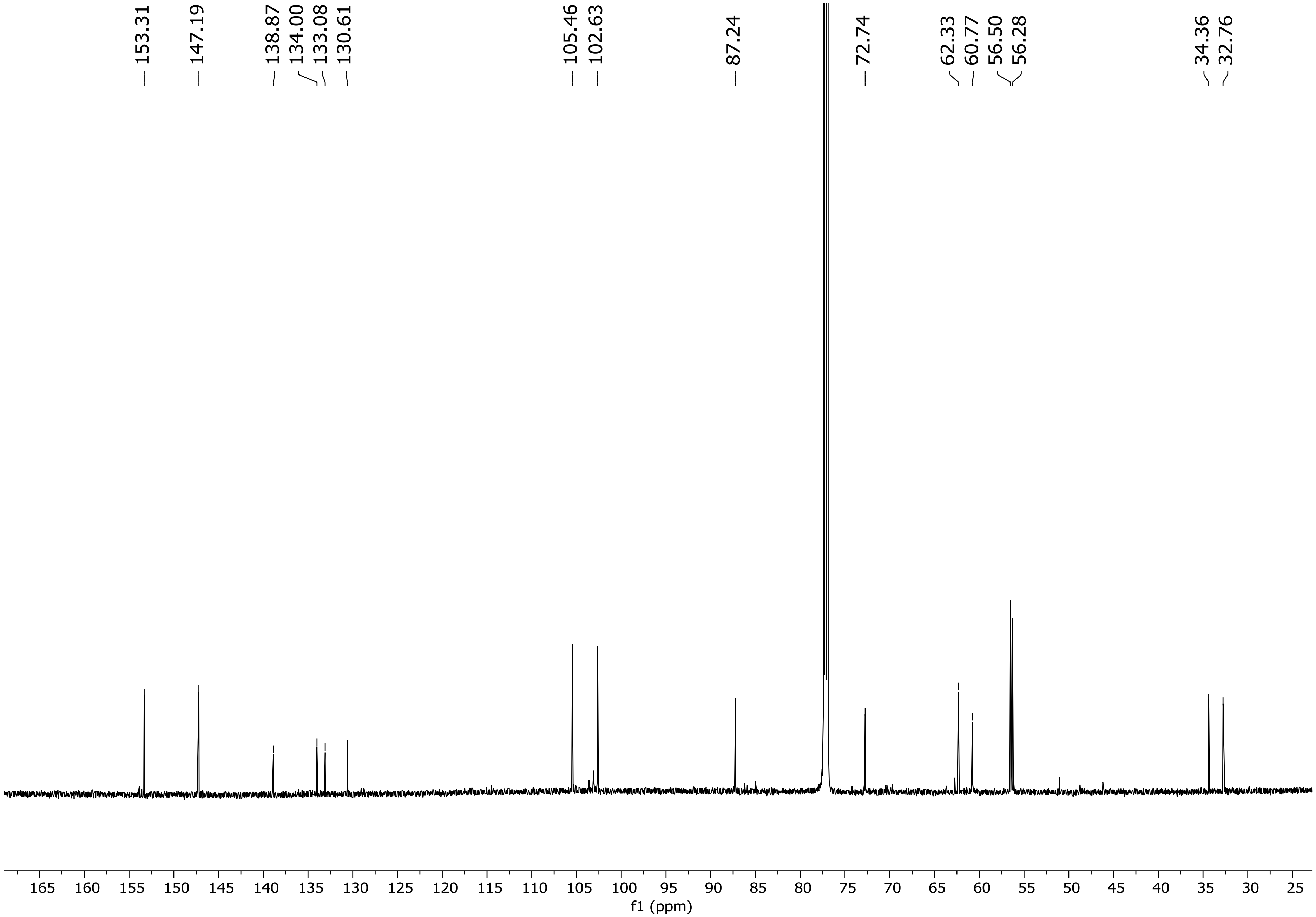

Supplement: Supplementary file 1 [file antioxidants-10-00551-s001.pdf]
